# Transient Receptor Potential Cation Channel Subfamily V Member 1 Expression Promotes Chemoresistance in Non-Small-Cell Lung Cancer

**DOI:** 10.3389/fonc.2022.773654

**Published:** 2022-03-25

**Authors:** Li Li, Cheng Chen, Qin Xiang, Songqing Fan, Tian Xiao, Yangchao Chen, Duo Zheng

**Affiliations:** ^1^ Guangdong Provincial Key Laboratory of Regional Immunity and Diseases, Shenzhen University International Cancer Center, Department of Cell Biology and Genetics, School of Medicine, Shenzhen University, Shenzhen, China; ^2^ Department of Pathology, The Second Xiangya Hospital, Central South University, Changsha, China; ^3^ School of Biomedical Sciences, Faculty of Medicine, The Chinese University of Hong Kong, Shatin, Hong Kong SAR, China

**Keywords:** TRPV1, cisplatin, fluorouracil, resistance, NSCLC

## Abstract

Approximately 85% of lung cancer cases are non-small-cell lung cancer (NSCLC). Chemoresistance is a leading cause of chemotherapy failure in NSCLC treatment. Transient receptor potential cation channel subfamily V, member 1 (TRPV1), a non-selective cation channel, plays multiple roles in tumorigenesis and tumor development, including tumor cell proliferation, death, and metastasis as well as the response to therapy. In this study, we found TRPV1 expression was increased in NSCLC. *TRPV1* overexpression induced cisplatin (DDP) and fluorouracil (5-FU) resistance in A549 cells independent of its channel function. TRPV1 expression was upregulated in A549-DDP/5-FU resistant cells, and DDP/5-FU sensitivity was restored by *TRPV1* knockdown. *TRPV1* overexpression mediated DDP and 5-FU resistance by upregulation of *ABCA5* drug transporter gene expression, thereby increasing drug efflux, enhancing homologous recombination (HR) DNA repair pathway to alleviate apoptosis and activating IL-8 signaling to promote cell survival. These findings demonstrate an essential role of TRPV1 in chemoresistance in NSCLC and implicate TRPV1 as a potential chemotherapeutic target.

## Introduction

Lung cancer is recognized as a serious public health concern worldwide, with 2 206 771 new cases diagnosed, and 1 796 144 deaths recorded in 2020. Lung cancer is the second most commonly diagnosed cancer and the leading cause of cancer death (1). Based on main histotype, prognostic, and therapeutic implications, lung carcinomas are categorized as small-cell lung carcinoma (SCLC) and non-small-cell lung cancer (NSCLC), with the latter accounting for approximately 85% of lung cancer cases (2, 3). Despite considerable progress in lung cancer therapy, the prognosis of lung cancer is poor, with a 5-year survival rate of only 10% to 20% in most countries (1). NSCLC therapy is limited by chemoresistance, which is mediated by multiple mechanisms, including mutation or loss of molecular targets, induction of autophagy, increased repair of DNA damage, inactivation of apoptosis, tumor heterogeneity and IL-6 and IL-8 release (4, 5). Therefore, novel molecular strategies for diagnosis and treatment are required urgently to improve the prognosis of NSCLC patients.

TRPV1, which is also known as the capsaicin receptor and the vanilloid receptor 1 (VR1), is a cation channel that is activated by various physical and chemical stimuli including capsaicin, temperatures exceeding 43°C, acidic conditions (pH <6) and vanilloids (6–8). Activation of the TRPV1 channel induces a flux of Ca^2+^ and Na^+^ ions into cells (9, 10). Intracellular Ca^2+^ and Na^+^ overload leads to cell death (11). TRPV1 is highly expressed in small-to-medium-sized neurons of the dorsal root, trigeminal and vagal ganglia (6, 12) and is associated with pain transduction (13). Over the last two decades, TRPV1 has been shown to play multiple roles in tumorigenesis and tumor development (14, 15). Altered TRPV1 expression has been identified in numerous cancer cell types. For example, TRPV1 upregulation was verified in human pancreatic cancer, prostate carcinoma, and breast cancer (16–18). Higher TRPV1 expression has also been reported in human primary brain tumors, and its expression correlates positively with the tumor grade (19). Moreover, some studies have demonstrated that TRPV1 suppresses cancer cell proliferation and induces cell death, while *TRPV1* overexpression prevents the proliferation of intestinal epithelial HCT116 cells, human pancreatic cancer PANC-1 cells, human skin carcinoma A431 cells and human melanoma A2058 and A375 cells (20–23). *TRPV1* overexpression was also found to promote apoptosis of human melanoma A2058 and A375 cells and breast cancer MCF-7 cells (22, 24). However, no report demonstrated the direct relationship between TRPV1 and NSCLC. Several studies merely showed that TRPV1 ligands had anti-cancer effect independent of TRPV1 in NSCLC. For example, TRPV1 agonists (*E*)-capsaicin or resiniferatoxin, and antagonists capsazepine or SB366791 induced apoptosis and necrosis in NSCLC H460 cells which there is no TRPV1 expression (25). Capsazepine analogs inhibited proliferation of H460 cells independent of TRPV1 (26). To date, there is no evidence to support a direct association between TRPV1 and chemotherapy resistance, although several studies have demonstrated the impact of TRPV1 agonists and antagonists on chemotherapy. DDP toxicity in breast cancer MCF-7 cells was increased by alpha-lipoic acid (ALA)-induced TRPV1 channel activation, and this effect was reversed by the TRPV1 blocker capsazepine (27). Capsaicin treatment increased the antiproliferative effects of pirarubicin, a major drug used in urinary bladder instillation chemotherapy (28). However, treatment with a combination of doxorubicin and the TRPV1 antagonist melatonin led to higher apoptosis rates than those induced by doxorubicin alone (29). In breast cancer MCF-7 cells, the toxicity of 5-FU was reduced by the TRPV1-channel inhibitor *Hypericum perforatum* (30). In short, the effect of TRPV1 on chemotherapy is unclear.

In this study, we demonstrated the following results: (1) the levels of TRPV1 is higher in NSCLC; (2) *TRPV1* overexpression induced DDP and 5-FU resistance in A549 cells independent of its channel function; (3) TRPV1 expression is increased in A549-DDP/5-FU resistant cells; (4) *TRPV1* knockdown reversed the DDP/5-FU resistance in A549-DDP resistant cells; (5) *TRPV1* overexpression induced DDP and 5-FU resistance by upregulation of the drug transporter *ABCA5*, enhancing DNA repair *via* the homologous recombination (HR) pathway, and increasing IL-8 release. Our findings clarify the roles of TRPV1 in DDP/5-FU resistance in NSCLC cells, and highlight the potential of TRPV1 as a novel prognostic factor for NSCLC progression and chemoresistance.

## Materials and Methods

### Molecular Cloning

For the generation of the pCDH-CMV-MCS-EF1-Puro-TRPV1-FLAG construct, the TRPV1-FLAG fragment was amplified by PCR using Q5^®^ High-Fidelity DNA Polymerase (New England BioLabs, Ipswich, MA, USA) according to the manufacturer’s instructions, and then inserted into the pCDH-CMV-MCS-EF1-Puro vector using *EcoR*I and *BamH*I restriction enzymes. For the generation of pLKO.1-TRPV1-shRNA-1, a TRPV1-shRNA-1 double-stranded oligonucleotide was generated using oligo_ TRPV1-shRNA-1_F (5’-CCG GGC TGC TGG CCT ATG TAA TTC TCT CGA GAG AAT TAC ATA GGC CAG CAG CTT TTT G-3’) and oligo_TRPV1-shRNA-1_R (5’-AAT TCA AAA AGC TGC TGG CCT ATG TAA TTC TCT CGA GAG AAT TAC ATA GGC CAG CAG C-3’) and then ligated with pLKO.1 using *EcoR*I and *Age*I restriction enzymes. For the generation of pLKO.1-TRPV1-shRNA-2, a TRPV1-shRNA-2 double-stranded oligonucleotide was generated using oligo_ TRPV1-shRNA-2_F (5’-CCG GAC GAG CAT GTA CAA TGA GAT TCT CGA GAA TCT CAT TGT ACA TGC TCG TTT TTT G-3’) and oligo_TRPV1-shRNA-2_R (5’-AAT TCA AAA AAC GAG CAT GTA CAA TGA GAT TCT CGA GAA TCT CAT TGT ACA TGC TCG T-3’) and then ligated with pLKO.1 using *EcoR*I and *Age*I enzymes. For the generation of the pCDH-CMV-MCS-EF1-Puro-IL-8-myc construct, the IL-8-myc fragment was amplified by PCR and then inserted into the pCDH-CMV-MCS-EF1-Puro vector using *Nhe*I and *BamH*I restriction enzymes. For the generation of pLKO.1-IL-8-shRNA-1, an IL-8-shRNA-1 double-stranded oligonucleotide was generated using oligo_IL-8-shRNA-1_F (5’-CCG GGG AGT GCT AAA GAA CTT AGA TCT CGA GAT CTA AGT TCT TTA GCA CTC CTT TTT-3’) and oligo_IL-8-shRNA-1_R (5’-AAT TAA AAA GGA GTG CTA AAG AAC TTA GAT CTC GAG ATC TAA GTT CTT TAG CAC TCC-3’) and then ligated with pLKO.1 using *EcoR*I and *Age*I restriction enzymes. For the generation of pLKO.1-IL-8-shRNA-2, an IL-8-shRNA-2 double-stranded oligonucleotide was generated using oligo_IL-8-shRNA-2_F (5’-CCG GGC ATA AAG ACA TAC TCC AAA CCT CGA GGT TTG GAG TAT GTC TTT ATG CTT TTT-3’) and oligo_IL-8-shRNA-2_R (5’-AAT TAA AAA GCA TAA AGA CAT ACT CCA AAC CTC GAG GTT TGG AGT ATG TCT TTA TGC-3’) and then ligated with pLKO.1 using *EcoR*I and *Age*I restriction enzymes.

### Cell Culture and Transfection

HEK293T, U2OS and A549 cells were cultured in Dulbecco’s modified Eagle’s medium (DMEM; HyClone, Cytiva, Marlborough, MA, USA) or RPMI 1640 (HyClone) supplemented with 10% fetal bovine serum (PAN-Seratech GmbH, Aidenbach, Germany) and 1% penicillin-streptomycin (Gibco, Thermo Fisher Scientific, Grand Island, NY, USA) at 37°C under 5% CO_2_. For transfection experiments, cells were seeded in 6-well plates (5×10^5^ cells/well) and incubated for 24 h before transfection with 2 μg plasmid DNA or 10 μM siRNA using Lipofectamine 3000 reagent (Invitrogen, Thermo Fisher Scientific, Grand Island, NY, USA).

### Stable Cell Line Establishment

*TRPV1* overexpression and *TRPV1* or *IL-8* knockdown cells were generated by infection with lentiviruses encoding TRPV1-FLAG, TRPV1 or IL-8 shRNAs. For production of the lentiviruses, the target plasmid was co-transfected into HEK293T cells with the pCMV delta R8.2 packaging plasmid and pCMV-VSV-G envelope plasmid. After 48 h, the supernatants were collected and filtered (pore size 0.45 μm). A549 cells were then infected with the viral supernatant fractions. At 48 h post-infection, the culture medium was replaced with fresh growth medium containing 2 μg/mL of puromycin for selection. The overexpression or knockdown efficiency was determined by qPCR, Western blotting or ELISA.

### Establishment of A549-DDP and A549-5-FU Resistant Cell Lines

The parental A549 cell line, which is sensitive to both DDP and 5-FU *in vitro*, was firstly incubated with 0.1 μM DDP (Sigma–Aldrich, Saint Louis, MO, USA) or 0.1 μg/mL 5-FU (Sigma–Aldrich). The concentrations of DDP and 5-FU was increased until the cells exhibited normal growth characteristics. Then, the A549 cells were treated continuously with gradually increasing concentrations of DDP (0.1–4 μM) or 5-FU (0.1–3.5 μg/mL) in 12 months to induce resistance to DDP or 5-FU.

### Quantitative Real-Time PCR

Total RNA was extracted from cells using TRIzol^®^ reagent (Invitrogen). Quantitative real-time PCR amplification was carried out using ChamQ Universal SYBR qPCR Master Mix (Vazyme, Nanjing, China) according to the manufacturer’s instructions. The CFX Connect Real-Time system (Bio-Rad Laboratories, Hercules, CA, USA) was used for all reactions. The following primers used: GAPDH (forward,5’-AGG TGA AGG TCG GAG TCA AC-3’; reverse, 5’- AGT TGA GGT CAA TGA AGG GG-3’), TRPV1 (forward,5’-TGG ATG GCT TGC CTC CCT TTA-3’; reverse, 5’-ACT GTA GCT GTC CAC AAA CAG-3’), IL-8 (forward, 5’-GCT CTG TGT GAA GGT GCA GT-3’; reverse, 5’-TGC ACC CAG TTT TCC TTG GG-3’), ABCA2 (forward, 5’-ACA CCT CTG GTT CTA CTC ACG G-3’; reverse, 5’-CCG ACA ATG TCT GCA CCA GTG A-3’), ABCA5 (forward, 5’-AGC TGT CAA AGG AAT GAG TGC-3’; reverse, 5’-TGC TGT TTG GCT TTG GGA TC-3’), ABCA8 (forward, 5’-ATC GGT TCC ATC CAA CAC CT-3’; reverse, 5’-GGT TGC ACA TCT TCC ACT GG-3’), ABCA12 (forward, 5’-CGG CAT TTC AGA TAC CAC CGT G-3’; reverse, 5’-CAG GAG TTG AGA TGC CAT TGG C-3’), ABCC5 (forward, 5’-GGC TGT ATT ACG GAA AGA GGC AC-3’; reverse, 5’-TCT TCT GTG AAC CAC TGG TTT CC-3’), ABCD1 (forward, 5’-CCT TCT GGA ACG CCT GTG GTA T-3’; reverse, 5’-TTC CAA GGC TGC CTT CTT CAC G-3’), TAP1 (forward, 5’-GCA GTC AAC TCC TGG ACC ACT A-3’; reverse, 5’-CAA GGT TCC CAC TGC TTA CAG C-3’).

### Cell Viability Assay

Drug sensitivity was determined using the Cell Counting Kit-8 (CCK-8) (Dalian Meliun Biotech Co., Ltd., Dalian, China) assay according to the manufacturer’s instructions. Briefly, cells were seeded in 96-well plates and viability was evaluated after incubation for 72 h with DDP/5-FU. Cells were pretreated with capsaicin (50 μM, HY-10448, MedChemExpress, Monmouth Junction, NJ, USA) for 15 min, capsazepine (2 μM, HY-15640, MedChemExpress) for 2 h, and EGTA (1 μM, 03777, Sigma–Aldrich) for 2 h.

### Colony Formation Assay

Cells were seeded in 6-well plates (500 cells/well for proliferation detection or 1000 cells/well for drug sensitivity detection) and cultured for 8–10 days until the colonies were observed clearly. The cells were fixed with 100% pre-cooled methanol for 15 min and then stained with 0.05% crystal violet for 15 min at room temperature (RT). The cells were washed with ddH_2_O and the colonies were counted, or the colonies area was quantified using AlphaView software.

### Immunofluorescent Staining

Cells were seeded onto 10-mm coverslips. After 24 h, the cells were transfected with 1 μg pCDH-CMV-MCS-EF1-Puro-TRPV1-FLAG or empty vector. After 24 h, the cells were treated with DDP or 5-FU for a further 24 h. Cells were fixed with 4% paraformaldehyde for 15 min at RT and then permeabilized using PBS containing 0.1% Triton X-100 for 15 min at RT. After permeabilization, cells were blocked with PBS containing 5% bovine serum albumin (BSA) for 30 min at RT. Cells were stained with anti-FLAG (1:500 dilution; F1804; Sigma–Aldrich) and anti-γH2AX (1:500 dilution; AP0099, ABclonal Technology Co.,Ltd., Wuhan, China) antibodies in the presence of 5% BSA for 1 h at RT. Cells were washed three times with PBS (5 min/wash). Cells were then stained with Alexa Fluor^®^ 488-AffiniPure Goat Anti-Mouse IgG (H+L) (1:400 dilution; 115-545-062; Jackson ImmunoResearch, West Grove, PA, USA) and Alexa Fluor^®^ 546 goat anti-rabbit IgG (H+L) (1:400 dilution; A11035; Invitrogen) antibodies at RT for 1 h. After washing five times with PBS (5 min/wash), nuclei were stained with DAPI (HY-D0814, MedChemExpress). The coverslips were mounted on glass slides with 5 μl mounting medium (Life Technologies, Thermo Fisher Scientific, Grand Island, NY, USA). Images were captured using a Nikon eclipse Ti2 confocal microscope (Japan).

### Western Blotting

Cells were lysed with SDS sample buffer containing 100 mM Tris-HCl (pH 6.8), 2% SDS, 10% glycerol (w/v), 5% β-mercaptoethanol, and 0.02% bromophenol blue. Cell lysates were subjected to sodium dodecyl sulfate-polyacrylamide gel electrophoresis (SDS-PAGE) and transferred to PVDF membranes for immunoblotting. Membranes were then incubated at 4°C overnight with the following primary antibodies: anti-FLAG (1:2,000; F1804; Sigma–Aldrich), anti-TRPV1 (1:1,000; ab111973; Abcam, Cambridge, UK), anti-β-actin (1:5,000; 66009-1-1g; Proteintech, Rosemont, IL, USA), anti-Myc (1:2,000; 2276; Cell Signaling Technology, Danvers, MA, USA), anti-β-tubulin (1:5,000; 10065-1-AP; Proteintech), anti-cleaved caspase 7 (1:1,000; 8438T; Cell Signaling Technology), anti-caspase 7 (1:1,000; 9492; Cell Signaling Technology), anti-cleaved caspase 9 (1:1,000; 7237T; Cell Signaling Technology), anti-caspase 9 (1:1,000; 9505S; Cell Signaling Technology), anti-cleaved caspase 3 (1:1,000; 9761T; Cell Signaling Technology), anti-caspase 3 (1:1,000; 9665T; Cell Signaling Technology), anti-p-p38 MAPK (T180/Y182) (1:1,000; 4511S; Cell Signaling Technology), anti-p38 MAPK (1:1,000; 8690S; Cell Signaling Technology) or anti-LC3II (1:1,000; 3868S; Cell Signaling Technology). Membranes were then incubated at RT for 2 h with the following secondary antibodies: peroxidase-conjugated AffiniPure goat anti-rabbit IgG (1:10,000; 111-035-008; Jackson ImmunoResearch) or peroxidase-conjugated AffiniPure goat anti-mouse IgG (1:10,000; 115-035-008; Jackson ImmunoResearch). Finally, immunoreactive bands were visualized by exposure of the membranes to Luminata crescendo Western HRP substrate (34577, Thermo Fisher Scientific) and imaged using the Fluor Chem Q system (protein Simple, CA, USA).

### Tissue Microarray (TMA) and Immunohistochemistry (IHC)

TMA sections containing tissues from 100 human patients with lung adenocarcinoma and 53 normal lung tissues were obtained from the Second Xiangya Hospital of Central South University (Changsha, China). IHC was then performed to examine the TRPV1 expression profile. Briefly, lung cancer clinical samples and normal lung tissue were fixed and embedded in paraffin, sectioned and stained with hematoxylin and eosin. IHC staining of the paraffin-embedded tumor tissues was performed with anti-TRPV1 (1:100; ab3487; Abcam, Cambridge, UK). TRPV1 staining was scored by two independent pathologists blinded to the patient characteristics.

Staining extent was based on the percentage of TRPV1 positive cells: ≤10% (0), 11-25% (1), 26-50% (2), 51-75% (3), and >75% (4), while staining intensity was classified as 0 (negative), 1 (weak), 2 (moderate) or 3 (strong). According to these scores, the results of IHC were classified as negative (-, score 0–1), weak (+, score 2–4), moderate (++, score 5–8), or strong (+++, score 9–12).

### Apoptosis

Cell apoptosis was detected using Annexin V-PE and the RedNucleus II Apoptosis Kit (A6079, US Everbright^®^ Inc., Suzhou, China) according to the manufacturer’s instructions. Briefly, cells were seeded in 6-well plates (4×10^5^ cells/well). After experimental treatment, the cells were collected and stained with Annexin V-PE and RedNucleus II for 20 min at RT and analyzed by flow cytometry using a CytoFLEX flow cytometer (Beckman Coulter, Brea, CA, USA).

### DR-GFP and EJ5-GFP Assay

U2OS DR-GFP or EJ5-GFP cells were transfected with empty vector or the pCDH-CMV-MCS-EF1-Puro-TRPV1-FLAG construct 24 h before HA-I-*Sce*I transfection. After 48 h, cells were harvested and analyzed by flow cytometry analysis using a CytoFLEX flow cytometer (Beckman Coulter). The percentage of GFP-positive cells as a measure of HR or non-homologous end-joining (NHEJ) DNA repair efficiency.

### ELISA

Culture supernatants were collected from cells grown in 12-well plates. Subsequently, 10-fold serial dilutions of the culture supernatants were prepared for analysis using IL-8 Human ELISA Kits (ab46032, Abcam) according to the manufacturer’s instructions.

### Xenografts in Nude Mice

Four to five weeks of male BALB/c athymic (NU/NU) mice were purchased from Beijing Vital River Laboratory Animal Technology Co., Ltd. All animal research procedures were performed according to the protocols of the Animal Care and Use Ethics Committee of Shenzhen University Health Science Center and all animals were treated in strict accordance with protocols approved by the Institutional Animal Use Committee of the Health Science Center, Shenzhen University. The mice were injected with 5 ×10^6^ A549-empty vector, A549- TRPV1-FLAG, A549 and A549-DDP cells. The mice were randomly divided into two groups after the diameter of the xenografted tumors had reached approximately 5 mm in diameter. The xenografted mice were then intraperitoneally administrated with 0.9% NaCl or DDP (3 mg/kg per day) for three times a week. The tumor volume was measured before every injection. Tumor volume was estimated as *V* = (*L* × *W* ^2^)/2 (*V*, volume; *L*, length; *W*, width).

### Statistical Analyses

Statistical analyses were performed using a two-tailed unpaired Student’s *t -*test. All experiments were performed at least three times. Results are presented as mean ± standard error of the mean (SEM). *P <* 0.05 was considered to indicate statistical significance.

## Results

### The Expression of TRPV1 Is Significantly Increased in NSCLC

**Figure 1:**
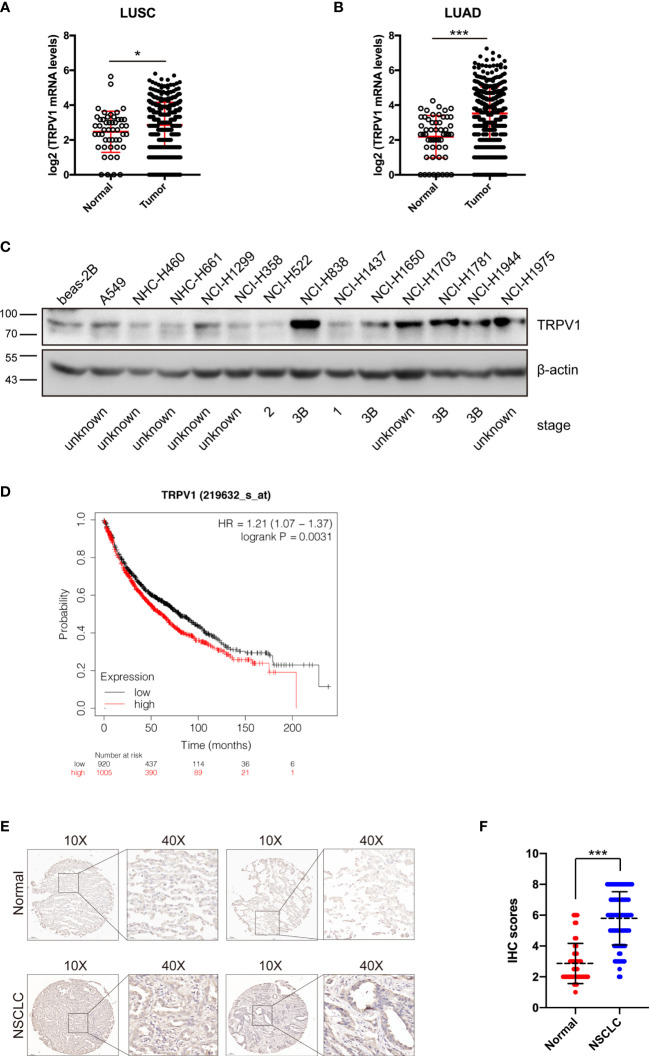
TRPV1 expression is increased in non-small-cell lung cancer. **(A)** *TRPV1* mRNA expression is increased in lung squamous carcinoma (LUSC) patients from TCGA database. Normal, n=49; Tumor, n=502. **(B)** The *TRPV1* mRNA expression level is increased in lung adenocarcinoma (LUAD) patients from TCGA database. Normal, n=59; Tumor, n=535. **(C)** Immunoblotting analysis of TRPV1 protein expression in NSCLC cell lines; β-actin served as the loading control. **(D)** Kaplan–Meier plots of lung cancer patients with different *TRPV1* mRNA expression (high versus low) in the KM-Plotter database (www.kmplot.com). (Analysis criteria: patients separated according to the follow-up threshold, all; optimal cutoff, auto select). **(E)** IHC staining showed the TRPV1 expression in NSCLC and normal lung tissues. Scale bar, 100 μm. **(F)** Statistical analysis of **(E)**. Three independent experiments were performed. Data represent mean ± SD; *P < 0.05. ***P < 0.001.

TRPV1 is expressed at high levels in human primary brain tumors, pancreatic cancer squamous cell carcinoma of the tongue, prostate carcinoma and breast cancer (16–19, 31). However, the expression profile of TRPV1 in NSCLC is unclear. To determine this, we evaluated *TRPV1* mRNA levels in TCGA database. This analysis showed that *TRPV1* mRNA expression was significantly increased in squamous cell carcinoma (LUSC) (**Figure 1A**) and adenocarcinoma (LUAD) (**Figure 1B**). Moreover, we detected TRPV1 protein expression in a panel of NSCLC cell lines, with normal human bronchial epithelium cells (beas-2B) as a control. Even if several clinical stage information cannot be found through searching ATCC database or public database such as Cellosaurus (https://web.expasy.org/cellosaurus/), however, compared with beas-2B cells, TRPV1 protein expression was higher in NSCLC cell lines isolated from late-stage patients such as NCI-H838, NCI-H1781 and NCI-H1944 (**Figure 1C**). Finally, we used the Kaplan–Meier plotter on-line database (http://kmplot.com/analysis) to explore the possible association of TRPV1 with NCSLC patient survival. Of 1925 patients, patients with the high *TRPV1* mRNA level expression (n = 1005) displayed worse survival than those with low *TRPV1* mRNA level expression (n = 920), suggesting that low expression of TRPV1 is more beneficial for NCSLC patients’ survival (**Figure 1D**). In addition, to evaluate the protein expression of TRPV1 in NSCLC clinical samples, we performed IHC on 100 clinical NSCLC specimens. Significantly increased level of TRPV1 was observed in clinical NSCLC tissues compared with normal lung tissues (**Figures 1E, F**). The association between TRPV1 protein expression and patient survival on these 100 clinical NSCLC samples was also analyzed. We found that patients with high TRPV1 expression have a trend toward poor prognosis, however, there is no statistical significance (**Figure S1**). Thus, these results revealed that TRPV1 expression is significantly increased in NSCLC, which was associated with poor survival in lung cancer patients.

### TRPV1 Is Involved in DDP and 5-FU Resistance in NSCLC Cells

**Figure 2:**
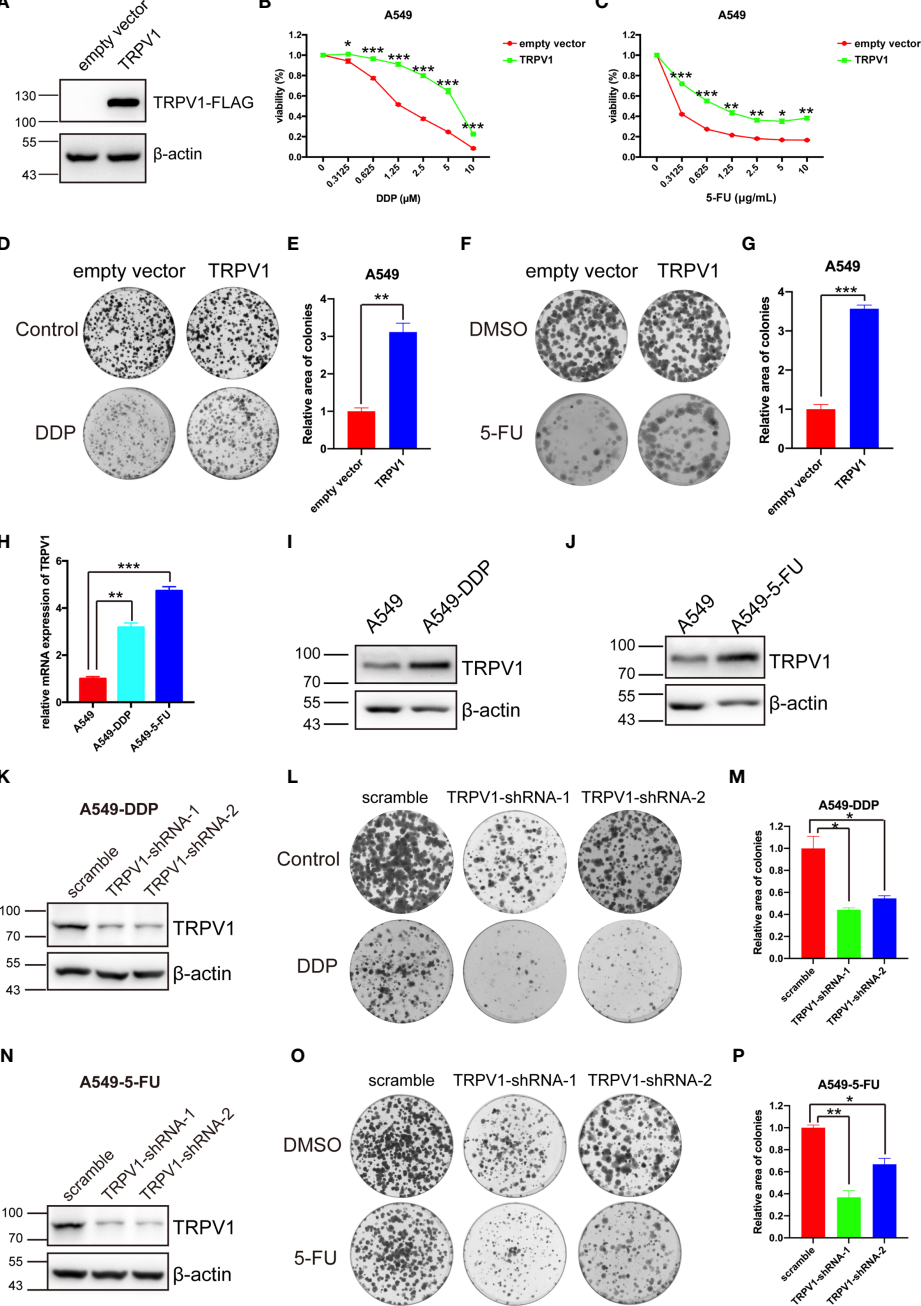
TRPV1 is associated with DDP and 5-FU sensitivity alteration. **(A)** *TRPV1* overexpression in A549 cells was confirmed by immunoblotting; β-actin served as the loading control. **(B)** CCK-8 assay showing that *TRPV1* overexpression decreased DDP sensitivity in A549 cells. Cells were treated for 72 h. **(C)** CCK-8 assay showing that *TRPV1* overexpression decreased 5-FU sensitivity in A549 cells. Cells were treated for 72 h. **(D)** Representative image of a colony formation assay of the effect of *TRPV1* overexpression on DDP sensitivity in A549 cells. Cells were treated with 0.5 μM DDP. **(E)** Statistical analysis of C. **(F)** Representative image of a colony formation assay of the effect of *TRPV1* overexpression on 5-FU sensitivity in A549 cells. Cells were treated with 0.15 μg/mL 5-FU. **(G)** Statistical analysis of **(F)**. **(H)** Quantitative PCR analysis showing that *TRPV1* mRNA expression is increased in A549-DDP/5-FU resistant cell lines. **(I)** Immunoblotting analysis of TRPV1 protein expression in A549-DDP resistant cell lines; β-actin served as the loading control. **(J)** Immunoblotting analysis of TRPV1 protein expression in A549-5-FU resistant cell lines; β-actin served as the loading control. **(K)** Immunoblotting analysis of *TRPV1* knockdown efficiency; β-actin served as the loading control.**(L)** Representative image of a colony formation assay of the effect of *TRPV1* knockdown on DDP sensitivity in A549-DDP resistance cells. Cells were treated with 4 μM DDP. **(M)** Statistical analysis of L. **(N)** Immunoblotting analysis of *TRPV1* knockdown efficiency; β-actin served as the loading control. **(O)** Representative image of a colony formation assay of the effect of *TRPV1* knockdown on 5-FU sensitivity in A549-5-FU resistance cells. Cells were treated with 3.5 μg/mL 5-FU. **(P)** Statistical analysis of O. Three independent experiments were performed. Data represent mean ± SEM; *P < 0.05. **P < 0.01. ***P < 0.001.

Chemotherapy is one of the main strategies used to treat lung cancer, although resistance to chemotherapy is an ongoing challenge because it limits the effectiveness of anticancer drugs. To investigate a potential association of TRPV1 with chemoresistance, we first established an A549 cell line stably overexpressing the TRPV1-FLAG fusion protein; the empty vector was used as a control. Successful expression of TRPV1-FLAG was confirmed by immunoblotting (**Figure 2A**). The CCK-8 and colony formation assays were performed using A549-TRPV1-FLAG and A549-empty-vector stable cell lines to explore the effects of *TRPV1* overexpression on the sensitivity to DDP and 5-FU. Both assays suggested that *TRPV1* overexpression decreased the sensitivity of A549 cells to DDP and 5-FU (**Figures 2B–G**). To investigate whether *TRPV1* overexpression decreased the sensitivity of A549 cells to DDP and 5-FU by promoting proliferation. The CCK-8 and colony formation assays were performed to explore the role of *TRPV1* overexpression on A549 cell proliferation. Both these two experiments results suggested that overexpression of *TRPV1* significantly attenuated the proliferative and clonogenic capacity in A549 cells (**Figures S2A–C**), which indicated that the decreased the sensitivity of A549 cells to DDP and 5-FU induced by *TRPV1* overexpression may not due to promoting proliferation. To confirm the protective effects of TRPV1 against DDP and 5-FU, we established DDP and 5-FU resistant cell lines and tested the resistance characteristics (**Figure S3**), and then detected the expression of TRPV1. Compared with parental A549 cells, we found that TRPV1 expression was upregulated at both the mRNA and protein levels in A549-DDP and A549-5-FU resistant cell lines (**Figures 2H–J**).

In addition, we used shRNAs to knock down *TRPV1* in A549-DDP and A549-5-FU resistant cells. Western blot analysis of the efficiency of *TRPV1* knockdown in A549-DDP and A549-5-FU resistant cells confirmed that TRPV1 protein levels were dramatically reduced in the A549-DDP-*TRPV1*-shRNA-1/2 and A549-5-FU-*TRPV1*-shRNA-1/2 stable cell lines (**Figures 2K, N**). Colony formation assays of the effect of *TRPV1* knockdown on DDP and 5-FU resistance suggested that *TRPV1* knockdown reversed the DDP and 5-FU resistance of A549-DDP (**Figures 2L, M**) and A549-5-FU (**Figures 2O, P**) resistant cells. Taken together, these results demonstrated the involvement of TRPV1 in DDP and 5-FU resistance in NSCLC cells.

### *TRPV1* Overexpression Induced DDP Resistance in a Mouse Xenograft Model

**Figure 3:**
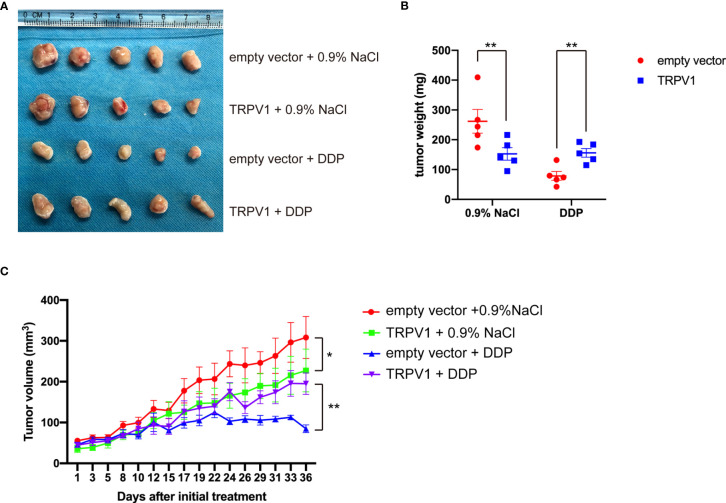
*TRPV1* overexpression induced DDP resistance in a mouse xenograft model. **(A)** Images of A549 empty vector and A549-TRPV1 xenografts treated with or without DDP. **(B)** The tumor weight of animals with A549 empty vector and A549-TRPV1 xenografts treated with or without DDP. **(C)** The tumor volume of A549 empty vector and A549-TRPV1 xenografts treated with or without DDP. Data represent mean ± SEM; *P < 0.05. **P < 0.01.

We next examined the effect of *TRPV1* overexpression on DDP resistance on A549 cell xenografts in mice. Compared with empty vector group, the tumor weight of TRPV1 overexpression group was reduced and the tumor growth was inhibited (**Figures 3A–C**). In present of DDP, the tumor weight and growth of TRPV1 overexpression group were significant increased compared with the empty vector group (**Figures 3A–C**), which indicated that *TRPV1* overexpression induced DDP resistance *in vivo*.

### *TRPV1* Overexpression Induces DDP and 5-FU Resistance Independent of Its Channel Function

**Figure 4:**
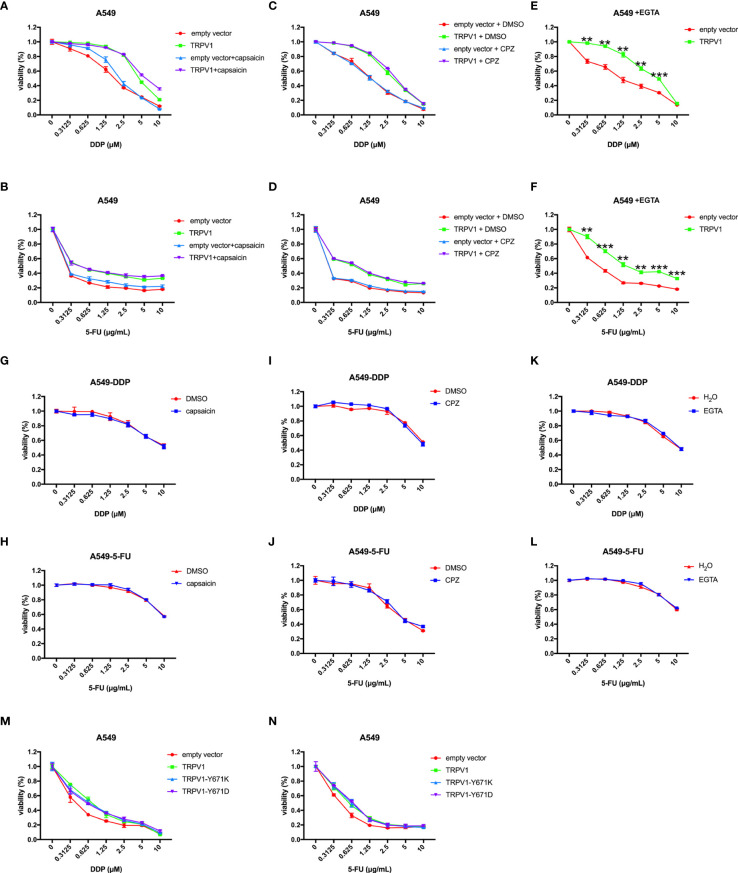
*TRPV1* overexpression induced DDP and 5-FU resistance independent of its channel function. **(A, B)** CCK-8 assay showing that capsaicin treatment has no effect on DDP or 5-FU sensitivity and resistance induced by *TRPV1* overexpression. **(C,D)** CCK-8 assay showing that capsazepine treatment has no effect on DDP or 5-FU sensitivity and resistance induced in A549 cells by *TRPV1* overexpression. **(E, F)** CCK-8 assay showing that *TRPV1* overexpression still decreased DDP and 5-FU sensitivity of A549 cells cultured in the presence of EGTA. **(G, H)** CCK-8 assay showing that capsaicin treatment has no effect on DDP or 5-FU sensitivity of A549-DDP/5-FU resistant cells. **(I, J)** CCK-8 assay showing that capsazepine treatment has no effect on DDP or 5-FU sensitivity of A549-DDP/5-FU resistance cells. **(K, L)** CCK-8 assay showing that EGTA treatment has no effect on DDP or 5-FU sensitivity of A549-DDP/5-FU resistant cells. **(M, N)** CCK-8 assay showing that *TRPV1* overexpression induced DDP or 5-FU resistance independent of the calcium signaling pathway. Three independent experiments were performed. Statistical significance was determined using Student’s *t* test. Data represent mean ± SEM. **P < 0.01. ***P < 0.001.

Since TRPV1 is a non-selective cation channel that can be activated to mediate cellular influx of Ca^2+^ and intracellular Ca^2+^ homeostasis is critical for survival, we then investigated the role of this function in the ability of TRPV1 to induce chemoresistance. Capsaicin is a well-known agonist of TRPV1, and it can active TRPV1 to increase intracellular Ca^2+^ concentration (32, 33). First, we evaluated the effect of pretreatment with capsaicin on DDP and 5-FU resistance induced by *TRPV1* overexpression. Capsaicin pretreatment had no effect on the DDP and 5-FU sensitivity and resistance induced by *TRPV1* overexpression (**Figures 4A, B**). Capsazepine is a well-known antagonist of TRPV1 and it can inhibit the Ca^2+^ influx (32, 34). Similarly, pretreatment with the TRPV1 antagonist capsazepine did not alter the DDP and 5-FU sensitivity and resistance induced by *TRPV1* overexpression (**Figures 4C, D**). Furthermore, capsaicin and capsazepine did not change the DDP and 5-FU resistance of the A549-DDP and A549-5-FU resistant cell lines (**Figures 4G–J**). These results suggested that activation/inhibition the TRPV1 ion channel by capsaicin/capsazepine had no effect on the chemoresistance induced by *TRPV1* overexpression.

We next sought to investigate whether the effect of *TRPV1* overexpression on chemoresistance is associated with the Ca^2+^ signaling pathway using EGTA to chelate extracellular Ca^2+^. In the absence of extracellular Ca^2+^, *TRPV1* overexpression still induced DDP and 5-FU resistance (**Figures 4E, F**). Moreover, EGTA pretreatment had no effect on the DDP and 5-FU resistance of the A549-DDP and A549-5-FU resistant cell lines (**Figures 4K, L**). To further address the implication of TRPV1 in chemoresistance-induced Ca^2+^ influx, we explored the impact of full-length TRPV1 WT, TRPV1-Y671K (a TRPV1 mutant with defective Ca^2+^ permeability) or TRPV1-Y671D (a TRPV1 mutant with normal Ca^2+^ permeability) overexpression in A549 cells. Overexpression of TRPV1-Y671K and TRPV1-Y671D decreased DDP and 5-FU sensitivity to the degree that was comparable to that of TRPV1 WT (**Figures 4M, N**).

These results indicated that TRPV1 induced chemoresistance *via* a mechanism that is independent of the Ca^2+^ signaling pathway.

### *TRPV1* Overexpression Induces DDP and 5-FU Resistance by Upregulation of Drug Transporters

A variety of factors contribute to drug resistance, including induction of autophagy, inactivation of apoptosis, increased repair of DNA damage and drug efflux and metabolism (4). To explore the mechanism of TRPV1-mediated DDP and 5-FU resistance, we performed RNA sequencing. Kyoto Genes and Genomes (KEGG) pathway enrichment analysis of the differentially expressed genes associated with *TRPV1* overexpression compared with the empty vector control revealed numerous enrichment-related pathways. Among the upregulated pathways, the top 15 pathways in *Homo sapiens* are listed (**Figure S4A**). Of Which, homologous recombination, nucleotide excision repair, non-homologous end-joining, mismatch repair, base excision repair and ABC transporters may involve in chemoresistance mediated by TRPV1. The significant pathways of differentially upregulated mRNAs in the TRPV1 overexpression + 5-FU group compared with the empty vector + 5-FU group are listed (**Figure S4B**). Among them, homologous recombination, nucleotide excision repair, non-homologous end-joining, base excision repair and ABC transporters may involve in 5-FU resistance mediated by TRPV1.

The ABC transporters *ABCA2*, *ABCA5*, *ABCA8*, *ABCA12*, *ABCC5*, *ABCD1* and *TAP1* were upregulated by *TRPV1* overexpression with or without 5-FU treatment. To determine whether these ABC transporters are involved in DDP/5-FU resistance induced by *TRPV1* overexpression, we performed quantitative PCR analysis of their expression in A549-DDP/5-FU resistant cells and A549 cells overexpressing TRPV1 and treated with or without DDP/5-FU.

**Figure 5:**
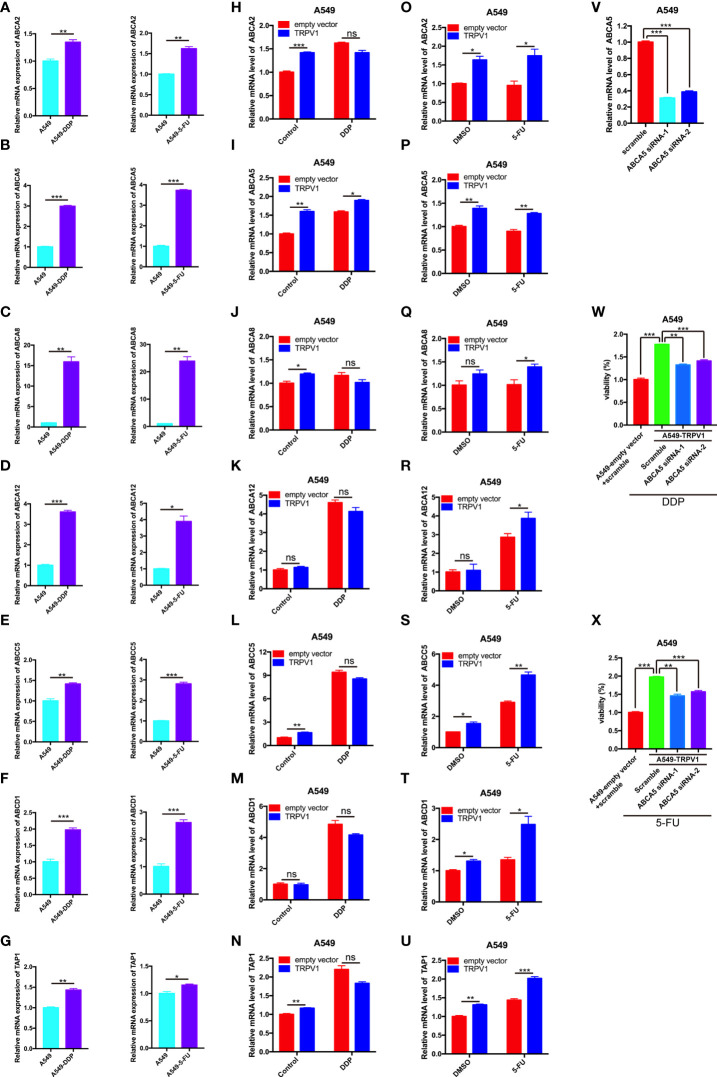
TRPV1 promotes DDP/5-FU resistance through upregulation of the expression of ABC drug transporters. **(A-G)** Quantitative PCR analysis of the expression of ABC transporter genes in A549 cells (azure bars) and A549-DDP/5-FU resistant cells (purple bars). **(H-U)** Quantitative PCR analysis of the expression of ABC transporter genes in A549 cells transfected with the empty vector (red bars) or stably overexpressing *TRPV1* (blue bars) and treated with or without DDP/5-FU. Cells were treated with DDP (1 μM) or 5-FU (0.5 μg/mL) for 24 h. **(V)** Quantitative PCR analysis of *ABCA5* knockdown efficiency. **(W)** CCK-8 assay of the effect of *ABCA5* knockdown on DDP resistance induced by *TRPV1* overexpression. *ABCA5* knockdown partially reversed DDP resistance induced by *TRPV1* overexpression. Cells were treated with 1.25 μM DDP for 72 h. **(X)** CCK-8 assay of the effect of *ABCA5* knockdown on 5-FU resistance induced by *TRPV1* overexpression. *ABCA5* knockdown partially reversed 5-FU resistance induced by *TRPV1* overexpression. Cells were treated with 0.3125 μg/mL 5-FU for 72 h. Three independent experiments were performed. Statistical significance was determined using Student’s *t* test. Data represent mean ± SEM. *P < 0.05. **P < 0.01. ***P < 0.001. ns, not statistically significant.

*ABCA2*, *ABCA5*, *ABCA8*, *ABCA12*, *ABCC5*, *ABCD1* and *TAP1* were expressed at very high levels in A549-DDP resistant cells and A549-5-FU resistant cells (**Figures 5A–G**). After *TRPV1* overexpression, the mRNA levels of *ABCA2*, *ABCA5*, *ABCA8*, *ABCC5* and *TAP1* were increased, while only *ABCA5* expression was increased in *TRPV1* overexpressing cells treated with DDP (**Figures 5H–N**). In the DMSO control group, *TRPV1* overexpression caused *ABCA2*, *ABCA5*, *ABCC5*, *ABCD1* and *TAP1* mRNA upregulation. However, following 5-FU treatment, the mRNA expression of *ABCA2*, *ABCA5*, *ABCA8*, *ABCA12*, *ABCC5*, *ABCD1* and *TAP1* was enhanced by *TRPV1* overexpression (**Figures 5O–U**). Only *ABCA5* upregulation was induced in cells overexpressing *TRPV1* and treated with DDP or 5-FU (**Figures 5I, P**).

To investigate the effect of *ABCA5* on the DDP and 5-FU resistance mediated by TRPV1, we analyzed the changes in DDP and 5-FU sensitivity following *TRPV1* overexpression in cells with *ABCA5* knockdown. CCK-8 assays showed that *ABCA5* knockdown partially reversed the DDP and 5-FU resistance mediated by *TRPV1* overexpression (**Figures 5W, X**). The efficiency of siRNA-mediated *ABCA5* knockdown was confirmed by quantitative PCR analysis (**Figure 5V**). These data indicated that the ABCA5 drug transporter regulates the DDP and 5-FU resistance mediated by *TRPV1* overexpression.

### *TRPV1* Overexpression Induces DDP and 5-FU Resistance by Enhancing HR DNA Repair

**Figure 6:**
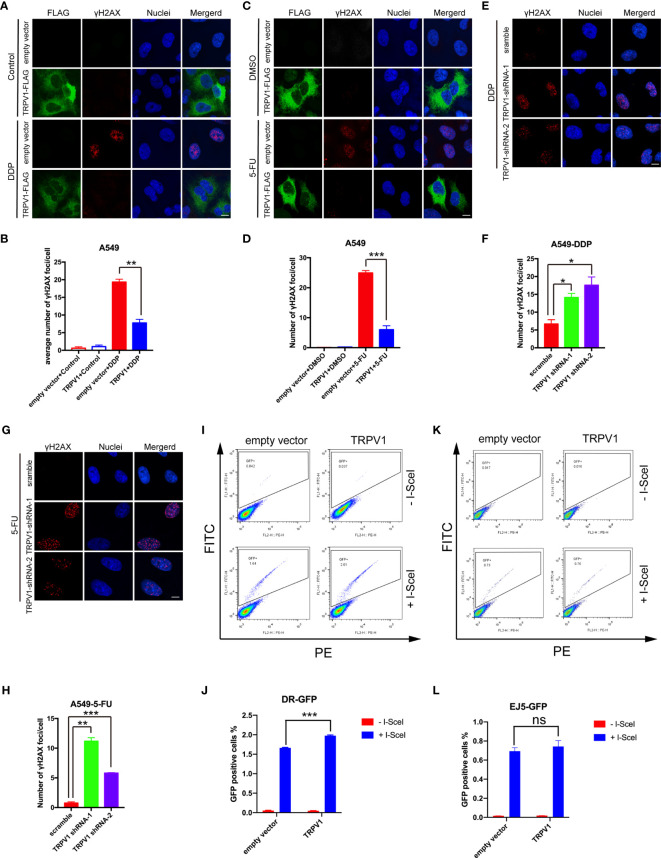
*TRPV1* overexpression increased DNA repair *via* the HR pathway. **(A)** *TRPV1* overexpression decreased the number of γH2AX foci induced in A549 cells by DDP treatment (1 μM) for 24 h. Scale bar, 10 μm. **(B)** Statistical analysis of A. At least 100 transfected cells were analyzed. **(C)** *TRPV1* overexpression decreased the number of γH2AX foci induced in A549 cells by 5-FU treatment (0.5 μg/mL) for 24 h. Scale bar, 10 μm. **(D)** Statistical analysis of C. At least 100 transfected cells were analyzed. **(E)** *TRPV1* knockdown increased the number of γH2AX foci induced in A549-DDP resistant cells by DDP treatment (4 μM) for 24 h. Scale bar, 10 μm. **(F)** Statistical analysis of E. At least 100 cells were analyzed. **(G)** *TRPV1* knockdown increased the number of γH2AX foci induced in A549-5-FU resistant cells by 5-FU treatment (3.5 μg/mL) for 24 h. Scale bar, 10 μm. **(H)** Statistical analysis of **(G)**. At least 100 cells were analyzed. **(I)** Flow cytometric DR-GFP reporter assay showing that *TRPV1* overexpression increased HR DNA repair efficacy. **(J)** Statistical analysis of I. **(K)** Flow cytometric EJ5-GFP reported assay showing that *TRPV1* overexpression had no effect on NHEJ DNA repair efficacy. **(L)** Statistical analysis of **(K)**. Three independent experiments were performed. Statistical significance was determined using Student’s *t* test. Data represent mean ± SEM; *P < 0.05. **P < 0.01. ***P < 0.001. ns, not statistically significant.

DDP and 5-FU treatment are known to cause DNA damage and numerous DNA repair pathways are involved in the responses to such treatment (35, 36). RNA sequencing analysis showed that *TRPV1* overexpression enriches genes involved in DNA repair with or without 5-FU treatment, indicating that *TRPV1* overexpression induces chemoresistance by enhancing DNA repair. To test this hypothesis, we examined the effect of *TRPV1* overexpression on γH2AX foci formation induced by DDP and 5-FU. These foci, which are formed by phosphorylation of the Ser-139 residue of H2AX, are recognized as markers of DNA damage (37). Confocal microscopy evaluation showed that *TRPV1* overexpression decreased the number of γH2AX foci induced by DDP and 5-FU (**Figures 6A–D**). Furthermore, *TRPV1* knockdown in A549-DDP/5-FU resistant cells increased the number of γH2AX foci induced by DDP and 5-FU (**Figures 6E–H**).

DNA double-strand breaks (DSBs) are repaired mainly by the HR and NHEJ pathways (38). To determine the involvement of TRPV1 in these pathways, we employed the DR-GFP and EJ5-GFP reporter systems to measure the HR and NHEJ activity, respectively, after *TRPV1* overexpression.

DR-GFP contains two differentially mutated green fluorescent protein (*GFP*) genes. Transient expression of the rare-cutting I-*Sce*I endonuclease produces a DSB in the upstream *GFP* gene. Functional GFP expression can only be restored by HR repair using the downstream *GFP* sequence and GFP-positive (GFP^+^) cells can be quantified by flow cytometry (39). The *TRPV1* construct or the empty vector were transfected into U2OS-DR-GFP cells harboring a single chromosomally integrated copy of the DR-GFP construct (40). After 24 h, the cells were transfected with I-*Sce*I. Flow cytometric analysis showed that the percentage of GFP^+^ cells was significantly increased in TRPV1 transfected cells after exogenous expression of I-*Sce*I, indicating an enhancement of the HR repair pathway after *TRPV1* overexpression (**Figures 6I, J**).

EJ5-GFP is composed of a promoter that is separated from a *GFP* open reading frame by a puro gene that is flanked by two I-*Sce*I sites in the same orientation. *GFP* can only be expressed after the puro gene is excised by NHEJ repair of the two I-*Sce*I-induced DSBs (41). In contrast to the results of the DR-GFP reporter assay, there were no significant differences in the percentage of GFP^+^ cells after *TRPV1* overexpression in U2OS cells stably transfected with the EJ5-GFP construct (**Figures 6K, L**).

These results suggested that *TRPV1* overexpression promotes DNA repair by HR rather than NHEJ.

### *TRPV1* Overexpression Alleviates Apoptosis Induced by DDP and 5-FU

**Figure 7:**
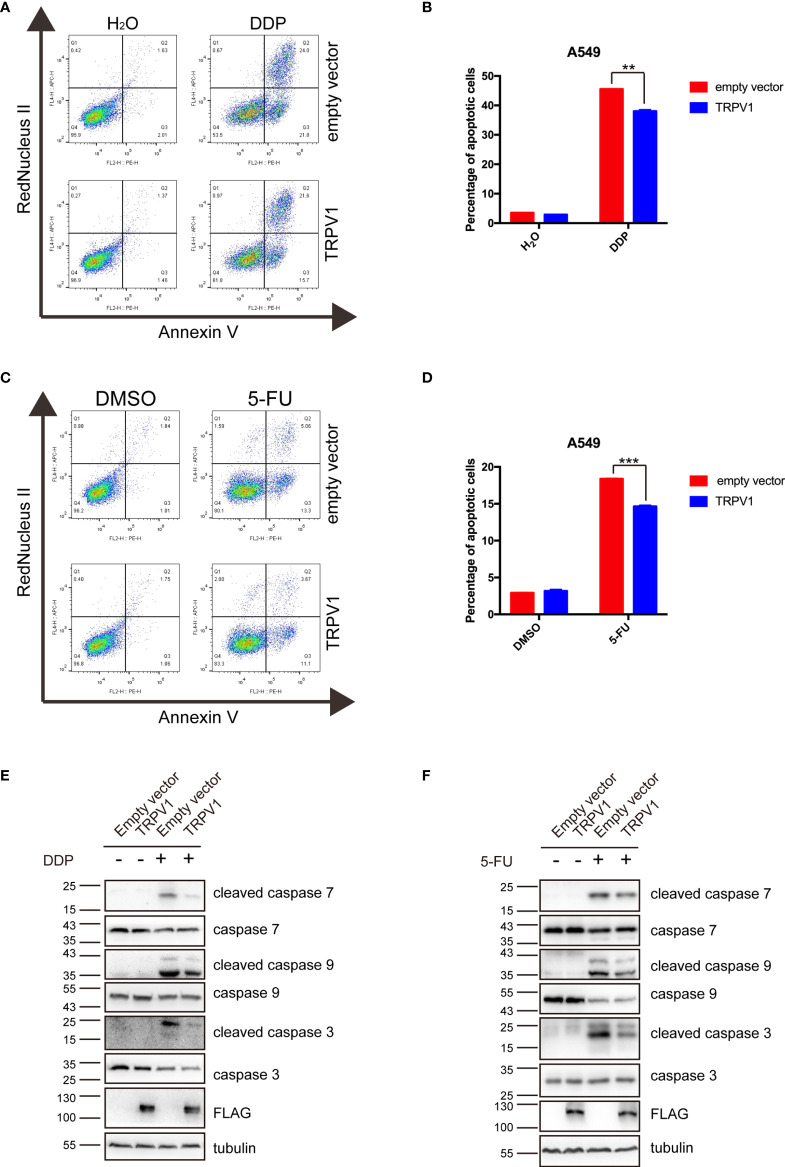
*TRPV1* overexpression alleviated apoptosis induced by DDP and 5-FU. **(A)** Flow cytometric analysis showing that *TRPV1* overexpression attenuated apoptosis induced by DDP treatment (20 μM) for 24 h. **(B)** Statistical analysis of **(A)**. **(C)** Flow cytometric analysis showing that *TRPV1* overexpression attenuated apoptosis induced by 5-FU treatment (160 μg/mL) for 24 h. **(D)** Statistical analysis of **(C)**. **(E)** Western blot analysis showing that *TRPV1* overexpression decreased the levels of cleaved caspase 7, 9 and 3 proteins induced by DDP treatment (20 μM) for 24 h; β-tubulin served as the loading control. **(F)** Western blot analysis showing that *TRPV1* overexpression decreased the levels of cleaved caspase 7, 9 and 3 proteins level induced by 5-FU treatment (160 μg/mL) for 24 h; β-tubulin served as the loading control. Three independent experiments were performed. Data represent mean ± SEM; **P < 0.01. ***P < 0.001.

DNA damage can trigger apoptosis (42). Repair of damaged DNA is important in preventing apoptosis. To determine the effect of *TRPV1* overexpression on apoptosis induced by DDP and 5-FU, we treated the A549-empty vector and A549-TRPV1 cells with DDP or 5-FU, and then determined the percentage of apoptotic cells by flow cytometry. Both the percentage of early and late apoptotic cells was analyzed, and the result suggested that *TRPV1* overexpression reduced the percentage of apoptotic cells stimulated by DDP and 5-FU treatment (**Figures 7A–D**).

To further investigate the effect of *TRPV1* overexpression on DDP/5-FU-mediated apoptosis, we evaluated caspase 3, 7 and 9 activation by Western blot analysis. *TRPV1* overexpression decreased the levels of cleaved caspase 3, 7 and 9 level stimulated by DDP (**Figure 7E**) and 5-FU (**Figure 7F**).

These results demonstrated that *TRPV1* overexpression alleviates the apoptosis induced by DDP and 5-FU.

### *TRPV1* Overexpression Induces DDP and 5-FU Resistance by Upregulation of IL-8 Expression

**Figure 8:**
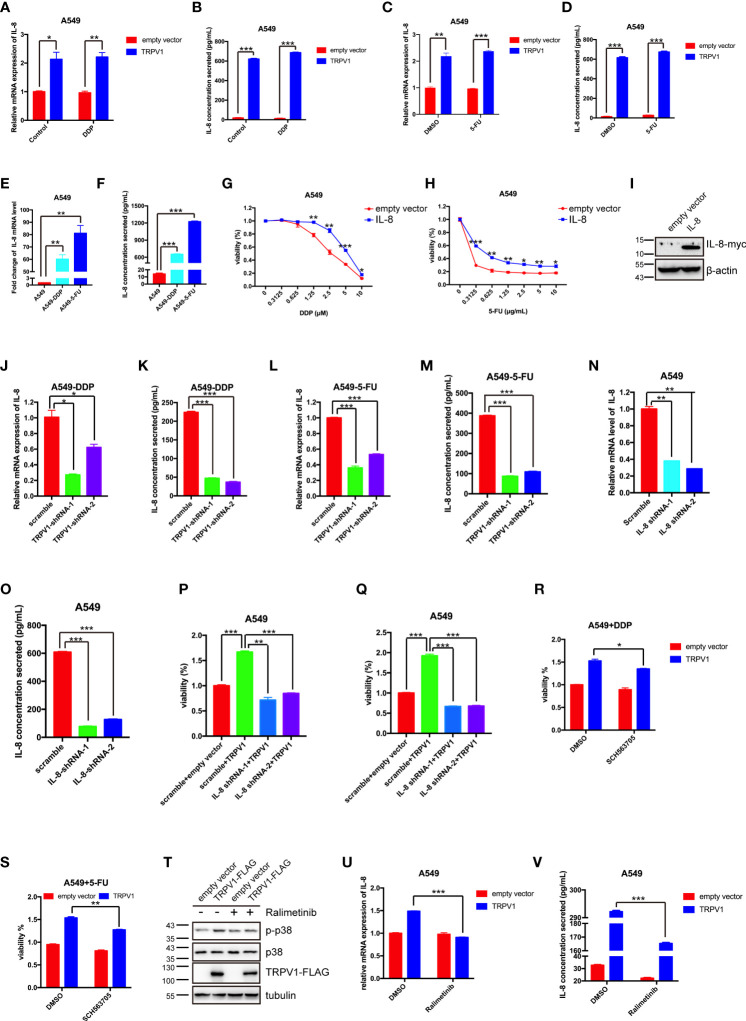
*TRPV1* overexpression induced DDP and 5-FU resistance through upregulation of IL-8 expression. **(A-D)** Quantitative PCR and ELISA analysis showing that *TRPV1* overexpression increased IL-8 mRNA expression and secretion with or without DDP treatment (1 μM) or 5-FU treatment (0.5 μg/mL) for 24 h. **(E, F)** Quantitative PCR and ELISA analysis showing that IL-8 mRNA expression and secretion were increased in A549-DDP and A549-5-FU resistant cells. **(G, H)** CCK-8 assay showing that *IL-8* overexpression decreased DDP and 5-FU sensitivity in A549 cells. **(I)** Western blot analysis showing IL-8 overexpression in A549 cells; β-actin served as the loading control. **(J–M)** Quantitative PCR and ELISA analysis showing that IL-8 mRNA expression and secretion were decreased in A549-DDP and A549-5-FU resistant cells after *TRPV1* knockdown. **(N, O)** Quantitative PCR and ELISA analysis of *IL-8* knockdown efficiency in A549 cells. **(P, Q)** CCK-8 assay showing that *IL-8* knockdown reversed DDP and 5-FU resistance induced by *TRPV1* overexpression. Cells were treated with DDP (2.5 μM) or 5-FU (0.625 μg/mL) for 72 h. **(R, S)** CCK-8 assay showing that inhibition of IL-8 receptors partially reversed DDP and 5-FU resistance induced by *TRPV1* overexpression. Cells were treated with DDP (1.25 μM)/5-FU (0.3125 μg/mL) and SCH563705 (20 μM) for 72 h. **(T)** Western blot analysis showing that *TRPV1* overexpression caused p38 activation. Cells were treated with ralimetinib (0.5μM) for 24 h. **(U, V)** Quantitative PCR and ELISA analysis showing that the upregulation of IL-8 mRNA expression and secretion level mediated by *TRPV1* overexpression were reduced by treatment with ralimetinib (0.5 μM) for 24h. Three independent experiments were performed. Statistical significance was determined using Student’s *t* test. Data represent mean ± SEM; *P < 0.05. **P < 0.01. ***P < 0.001.

RNA sequencing analysis showed that *IL-8* mRNA expression was dramatically upregulated by *TRPV1* overexpression with or without 5-FU treatment. *IL-8* knockdown has been reported to abrogate chemoresistance of docetaxel in breast cancer cells (5). Therefore, we hypothesized that *TRPV1* overexpression induces DDP/5-FU resistance *via* upregulation of IL-8 expression. First, we evaluated the IL-8 mRNA and protein expression after *TRPV1* overexpression in A549 cells by quantitative PCR and ELISA, respectively. *TRPV1* overexpression upregulated IL-8 expression at both the RNA and protein levels in the presence and absence of DDP/5-FU (**Figures 8A–D**). In addition, higher mRNA and protein expression of *IL-8* was detected in A549-DDP and A549-5-FU resistant cells compared with A549 parental cells (**Figures 8E, F**). To investigate the function of the high IL-8 expression in chemoresistance, we investigated the effect of *IL-8* overexpression on DDP/5-FU sensitivity of A549 cells using CCK-8 assay. *IL-8* overexpression decreased the sensitivity of A549 cells to DDP (**Figure 8G**) and 5-FU (**Figure 8H**). *IL-8* overexpression was confirmed by Western blotting (**Figure 8I**).

Next, we sought to confirm that the upregulation of IL-8 expression in A549-DDP and A549-5-FU resistant cells is due to the high expression of TRPV1. Following knockdown of *TRPV1* in A549-DDP and A549-5-FU resistant cells, quantitative PCR and ELISA analyses showed that IL-8 expression was decreased at both the mRNA and protein levels (**Figures 8J–M**). These results indicated that TRPV1 mediated IL-8 upregulation in A549-DDP and A549-5-FU resistant cells.

We next assessed the effect of *IL-8* knockdown on DDP and 5-FU resistance induced by *TRPV1* overexpression. CCK-8 assays suggested that *IL-8* knockdown abolished the DDP and 5-FU resistance induced by *TRPV1* overexpression (**Figures 8P, Q**). The *IL-8* knockdown efficiency after shRNA transfection was confirmed by quantitative PCR and ELISA (**Figures 8N, O**). To further determine the influence of IL-8 signaling of DDP/5-FU resistance induced by *TRPV1* overexpression, we employed an IL-8 receptor A/B antagonist SCH563705, CCK-8 assays showed that blockade of IL-8 A/B receptors partially reversed DDP and 5-FU resistance induced by TRPV1 overexpression (**Figures 8R, S**). Thus, we confirmed that inhibition of IL-8 A/B receptors can reverse the acquisition of chemoresistance mediated by *TRPV1* overexpression.

It has been reported that capsaicin-stimulated activation of TRPV1 induced IL-8 release through p38 MAPK signaling in human corneal epithelial cell (43). In this study, we found that *TRPV1* overexpression enhanced p38 phosphorylation, while the p38 MAPK inhibitor ralimetinib abolished the p38 activation caused by *TRPV1* overexpression (**Figure 8T**). Ralimetinib treatment also reversed the upregulation of IL-8 mRNA expression and protein secretion mediated by *TRPV1* overexpression (**Figures 8U, V**). These results indicated that the TRPV1-induced upregulation of IL-8 expression is mediated *via* p38 MAPK signaling.

Taken together, our results demonstrated that *TRPV1* overexpression stimulates p38 MAPK activation and leads to IL-8 expression upregulation, finally resulting in DDP and 5-FU resistance.

## Discussion

In present study, we aim to investigate the role and mechanism of TRPV1 in chemoresistance in NSCLC. we first showed that TRPV1 is upregulated significantly in NSCLC. We also demonstrated that TRPV1 overexpression decreased the sensitivity of NSCLC cells to DDP and 5-FU *via* a mechanism that is independent of its channel activity and the Ca^2+^ signaling pathway. Overall, we conclude that TRPV1 mediates DDP and 5-FU resistance through upregulation drug transporter ABCA5 expression, activation of the HR DNA repair pathway and increase of IL-8 expression.

To date, the expression and role of TRPV1 in NSCLC has not been elucidated. In this study, we discovered that TRPV1 expression is significantly upregulated in NSCLC, thus providing further evidence that altered TRPV1 expression in cancer is tumor-type specific. Our analysis of *TRPV1* mRNA levels in the TCGA database showed that TRPV1 expression is increased in human NSCLC. Moreover, TRPV1 expression correlates negatively with survival, implying that *TRPV1* might act as an oncogene in NSCLC. Simultaneously, the protein level of TRPV1 is upregulated in NSCLC clinical samples, and high expression of TRPV1 showed a tendency towards a poor prognosis, which indicated that TRPV1 expression could contribute to NSCLC progression. There is some evidence associating TRPV1 expression with the efficiency of radiotherapy and chemotherapy, and several investigators have reported the impact of TRPV1 agonists and antagonists on these treatments. For example, the TRPV1 channel inhibitors capsazepine, SB366791, AMG9810, and BCTC were reported to increase γ-ray-induced DNA damage in A549 cells, thus indicating that TRPV1 antagonists may increase the efficacy of radiotherapy (44). Capsaicin enhanced the antiproliferative effects of pirarubicin by activating TRPV1 in human bladder transitional cell carcinoma 5637 cells (28). The toxicity of DDP was increased by ALA-mediated TRPV1-channel activation in breast cancer MCF-7 cells (27). The TRPV1 antagonist melatonin increased the pro-apoptotic efficacy of doxorubicin in human breast cancer MCF-7 cells (29). However, in the same cell line, the TRPV1-channel inhibitor *Hypericum perforatum* reduced the toxicity of 5-FU (30). These studies suggested that TRPV1 agonists act synergistically with chemotherapeutic drugs in cancer therapy, while the roles of TRPV1 antagonists are controversial. In the present study, we demonstrated a direct association between TRPV1 expression and chemoresistance. We found that *TRPV1* overexpression induced DDP and 5-FU resistance *in vitro* and *in vivo*. Furthermore, TRPV1 expression was upregulated in A549-DDP and A549-5-FU resistant cells, and *TRPV1* knockdown re-sensitized resistant cells to DDP and 5-FU. This evidence verified the role of TRPV1 in promoting DDP/5-FU-resistance in A549 cells. However, TRPV1 activation or inhibition did not alter the degree of DDP/5-FU-resistance induced by *TRPV1* overexpression. In fact, the effects of TRPV1 agonists/antagonists on cancer have been found to be highly complex, in that both agonists and antagonists may possess anti-cancer effects, and the effects may function *via* TRPV1 or independently of TRPV1 (45). Moreover, removal of extracellular Ca^2+^ or blockade of the Ca^2+^ permeability of TRPV1 also had no effect on the degree of DDP/5-FU-resistance induced by *TRPV1* overexpression. In addition, we found TRPV1 localized in endoplasmic reticulum (**Figure S5**), which is consistent with the previous reports (46–48). Therefore, we conclude that TRPV1 induces chemoresistance independently of its ion channel function.

Our RNA sequencing analysis indicated that *TRPV1* overexpression induced chemoresistance through upregulation of the expression of ABC drug transporters and IL-8, and enhanced DNA repair. The ABC transporters mediate drug efflux from cells; thus, upregulation of ABC transporters contributes to the development of multidrug resistance (MDR). DDP is transported by ABCC2 and ABCB5 (49, 50), while ABCC1 mediates the efflux of 5-FU (51). However, *TRPV1* overexpression had no effect on the expression of *ABCC1*, *ABCB5* and *ABCC2* in our study (data not shown). In contrast, we showed that expression of *ABCA2*, *ABCA5*, *ABCA8*, *ABCA12*, *ABCC5*, *ABCD1* and *TAP1* was increased dramatically in A549-DDP/5-FU resistant cells. Moreover, we found that *TRPV1* overexpression elevated the expression of *ABCA5* with or without DDP and 5-FU treatment, and *ABCA5* knockdown partially reversed the DDP and 5-FU resistance induced by *TRPV1* overexpression. ABCA5 was reported to be expressed at high levels in mouse lung (52). Thus, TRPV1 is implicated as a key protein that regulates ABCA5 expression and DDP and 5-FU resistance in A549 cells.

DNA repair *via* the HR pathway plays a crucial role in maintaining cell survival following exposure to DNA-damaging agents (53). In the present study, we first found that *TRPV1* overexpression reduced the number of γH2AX foci induced by DDP and 5-FU in sensitive cells, while the number was increased by *TRPV1* knockdown in resistant cells. These data indicated that TRPV1 confers chemoresistance through its role in DNA repair. The ability of pretreatment with the TRPV1 channel inhibitors capsazepine, SB366791, AMG9810, and BCTC to increase γ-ray-induced DNA damage in A549 cells, indicates that TRPV1 antagonists may serve as radiosensitizers (44). However, whether these antagonists function through TRPV1 is unknown. In addition, TRPV1 channel activity is not involved in chemoresistance in our model. We also demonstrated that TRPV1 plays a positive role in DNA repair through HR rather than NHEJ. Thus, we identified a novel function of TRPV1 in HR DNA repair and chemotherapeutic drug sensitivity.

In this study, we also demonstrated that IL-8 expression was increased in A549-DDP/5-FU resistant cells, and *IL-8* overexpression induced DDP and 5-FU resistance. This result is consistent with previous studies on the association between IL-8 and chemoresistance. For instance, IL-8 expression is higher in the DDP resistant ovarian cancer lines PEA2 and PEO23, and *IL-8* knockdown re-sensitized resistant cell lines to DDP (54). IL-8 secretion is significantly increased in 5-FU-resistant human colorectal carcinoma cells HCT116 (55). IL-8 was found to be highly expressed in cancer-associated fibroblasts in the gastric cancer tissues of chemoresistant patients, and IL-8 was shown to promote DDP resistance in the human gastric cancer cell lines AGS and MGC-803 (56). Importantly, *TRPV1* overexpression elevated IL-8 mRNA expression and protein secretion in A549 cells. Furthermore, high expression of IL-8 in A549-DDP and A549-5-FU resistant cells was shown to be caused by upregulated TRPV1 expression. Additionally, depletion of IL-8 reversed the DDP/5-FU resistance induced by TRPV1 overexpression. This evidence confirms that TRPV1 overexpression confers DDP/5-FU resistance by upregulating IL-8 expression. The biological functions of IL-8 are mediated *via* two receptors, CXCR1 and CXCR2. Several previous studies have demonstrated that IL-8 receptors play a role in chemoresistance (54, 55). Therefore, we continued to investigate the roles of the CXCR1/2 in TRPV1-mediated chemoresistance. In accordance with previous reports, we found that inhibition of CXCR1/2 partially eliminated the DDP/5-FU resistance induced by TRPV1. Simultaneously, we investigated the mechanism underlying the role of TRPV1 in the upregulation of IL-8 expression. One study showed that capsaicin activated TRPV1 to induce IL-8 release *via* the MAPK signaling pathway in human corneal epithelial cells (43). Our RNA sequencing analysis also showed that TRPV1 overexpression activated MAPK signaling. Further investigations indicated that *TRPV1* overexpression activated the p38 MAPK pathway in A549 cells. Thus, our results demonstrate a link between DDP/5-FU resistance mediated by TRPV1 and enhanced IL-8 signaling activity.

Autophagy is one of the most important mechanisms underlying the resistance of cancer cells to chemotherapeutic agents. Suppression of autophagy with inhibitors or knockdown of autophagy-related genes increases the sensitivity of cancer cells to drugs (57). For instance, knockdown of *Atg7* enhances the sensitivity of acute myeloid leukemia to cytarabine and idarubicin (58). Inhibition of autophagy by 3-MA enhances the therapeutic efficacy of DDP and 5-FU in esophageal squamous cell carcinoma and colon cancer, respectively (59, 60). It has been reported that thymocytes from TRPV1 knockout mice display autophagy dysfunction, indicating that TRPV1 plays a positive role in this process (61). In accordance with this finding, we showed that *TRPV1* overexpression increased autophagy (**Figures S6A, B**). However, suppression of autophagy with the inhibitors chloroquine (CQ) and 3-MA did not reverse the DDP and 5-FU resistance mediated by *TRPV1* overexpression (**Figures S6C–F**). Thus, we conclude that *TRPV1* overexpression induces chemoresistance independently of autophagy.

**Figure 9:**
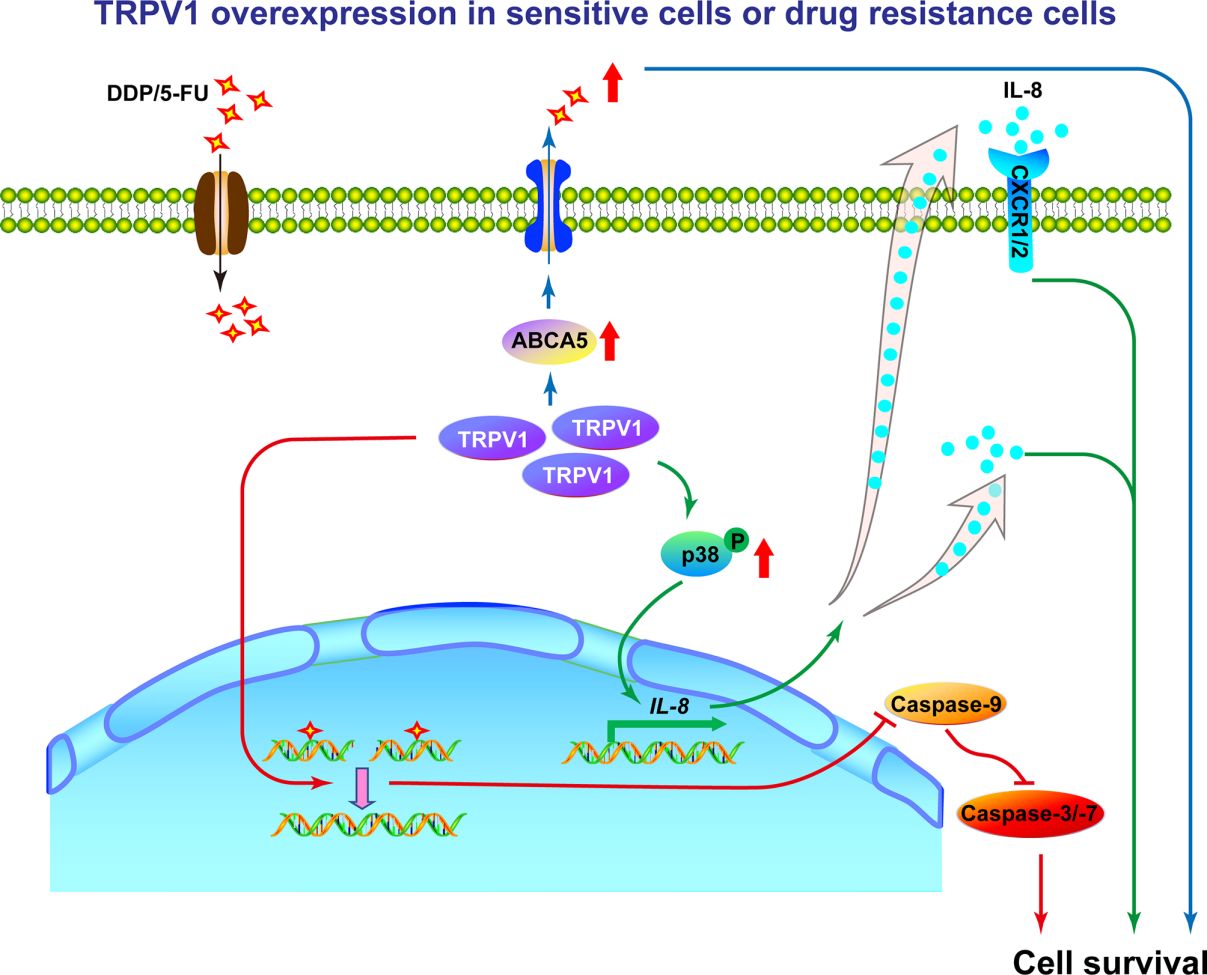
Working model of the mechanism by which TRPV1 regulates chemoresistance in A549 cells. In sensitive NSCLC cells, DDP or 5-FU induces DNA damage, resulting in cell death. In DDP/5-FU sensitive cells with *TRPV1* overexpression and in DDP/5-FU resistant cells, high levels of TRPV1 increase ABCA5 drug transporter expression resulting in increased drug efflux, enhances HR DNA repair efficiency subsequently leading to cell apoptosis inhibition, activates the p38 MAPK signaling pathway and increases IL-8 secretion to promote cell survival. Through the above three ways, TRPV1 finally induces chemoresistance. In addition, partial of IL-8 induces chemoresistance *via* CXCR1/2 receptors.

In conclusion, we show that TRPV1 is expressed at high levels in lung cancer and A549-DDP/5-FU resistant cells. Furthermore, TRPV1 overexpression induced chemoresistance through upregulation of the ABCA5 drug transporter, enhancement of HR DNA repair and activation of the p38 MAPK signaling pathway to increase IL-8 release in lung cancer A549 cells (**Figure 9**). Our study provides further clarification of the role of TRPV1 in modulating the sensitivity of lung cancer cells to chemotherapeutic agents and highlights its potential as a novel therapeutic target for lung cancer.

## Data Availability Statement

The original contributions presented in the study are included in the article/**Supplementary Material**. Further inquiries can be directed to the corresponding author.

## Ethics Statement

The studies involving human participants were reviewed and approved by The Ethics Review Committee of Central South University, The Second Xiangya Hospital. The patients/participants provided their written informed consent to participate in this study. The animal study was reviewed and approved by Animal Care and Use Ethics Committee of Shenzhen University Health Science Center.

## Author Contributions

DZ and LL conceived the idea. LL designed the experiments. LL, CC, QX, SF, and TX performed the experiments. LL and CC carried out statistical analysis. LL and DZ wrote/reviewed the paper. DZ and YC oversaw the research project. All authors contributed to the article and approved the submitted version.

## Funding

The present study was supported by grants from the National Natural Science Foundation of China (81802280, 81372149), the Shenzhen Municipal Government of China (JCYJ20180507182427559, JCYJ20210324093408024), the Natural Science Foundation of Guangdong Province (2019A1515010210, 2021A1515011046), the Guangdong Provincial Science and Technology Program (2019B030301009), and Shenzhen Key Medical Discipline Construction Fund (No. SZXK060).

## Conflict of Interest

The authors declare that the research was conducted in the absence of any commercial or financial relationships that could be construed as a potential conflict of interest.

## Publisher’s Note

All claims expressed in this article are solely those of the authors and do not necessarily represent those of their affiliated organizations, or those of the publisher, the editors and the reviewers. Any product that may be evaluated in this article, or claim that may be made by its manufacturer, is not guaranteed or endorsed by the publisher.

## References

[1] Sung H Ferlay J Siegel RL Laversanne M Soerjomataram I Jemal A. Global Cancer Statistics 2020: GLOBOCAN Estimates of Incidence and Mortality Worldwide for 36 Cancers in 185 Countries. CA Cancer J Clin (2021) 71(3):209–49. doi: 10.3322/caac.21660 33538338

[2] Herbst RS Heymach JV Lippman SM. Lung Cancer. N Engl J Med (2008) 359(13):1367–80. doi: 10.1056/NEJMra0802714 PMC10662965 18815398

[3] Rodriguez-Canales J Parra-Cuentas E Wistuba II. Diagnosis and Molecular Classification of Lung Cancer. Cancer Treat Res (2016) 170:25–46. doi: 10.1007/978-3-319-40389-2_2 27535388

[4] Sui X Chen R Wang Z Huang Z Kong N Zhang M. Autophagy and Chemotherapy Resistance: A Promising Therapeutic Target for Cancer Treatment. Cell Death Dis (2013) 4:e838. doi: 10.1038/cddis.2013.350 24113172 PMC3824660

[5] Su S Chen J Yao H Liu J Yu S Lao L. CD10(+)GPR77(+) Cancer-Associated Fibroblasts Promote Cancer Formation and Chemoresistance by Sustaining Cancer Stemness. Cell (2018) 172 841-856(4):e816. doi: 10.1016/j.cell.2018.01.009 29395328

[6] Caterina MJ Schumacher MA Tominaga M Rosen TA Levine JD Julius D. The Capsaicin Receptor: A Heat-Activated Ion Channel in the Pain Pathway. Nature (1997) 389(6653):816–24. doi: 10.1038/39807 9349813

[7] Tominaga M Caterina MJ Malmberg AB Rosen TA Gilbert H Skinner K. The Cloned Capsaicin Receptor Integrates Multiple Pain-Producing Stimuli. Neuron (1998) 21(3):531–43. doi: 10.1016/s0896-6273(00)80564-4 9768840

[8] Jordt SE Tominaga M Julius D. Acid Potentiation of the Capsaicin Receptor Determined by a Key Extracellular Site. Proc Natl Acad Sci USA (2000) 97(14):8134–9. doi: 10.1073/pnas.100129497 PMC16682 10859346

[9] Olah Z Szabo T Karai L Hough C Fields RD Caudle RM. Ligand-Induced Dynamic Membrane Changes and Cell Deletion Conferred by Vanilloid Receptor 1. J Biol Chem (2001) 276(14):11021–30. doi: 10.1074/jbc.M008392200 11124944

[10] Pecze L Blum W Schwaller B. Mechanism of Capsaicin Receptor TRPV1-Mediated Toxicity in Pain-Sensing Neurons Focusing on the Effects of Na(+)/Ca(2+) Fluxes and the Ca(2+)-Binding Protein Calretinin. Biochim Biophys Acta (2013) 1833(7):1680–91. doi: 10.1016/j.bbamcr.2012.08.018 22982061

[11] Pecze L Viskolcz B Olah Z. Molecular Surgery Concept From Bench to Bedside: A Focus on TRPV1+ Pain-Sensing Neurons. Front Physiol (2017) 8:378. doi: 10.3389/fphys.2017.00378 28626428 PMC5455100

[12] Helliwell RJ McLatchie LM Clarke M Winter J Bevan S McIntyre P. Capsaicin Sensitivity is Associated With the Expression of the Vanilloid (Capsaicin) Receptor (VR1) mRNA in Adult Rat Sensory Ganglia. Neurosci Lett (1998) 250(3):177–80. doi: 10.1016/s0304-3940(98)00475-3 9708861

[13] Patapoutian A Tate S Woolf CJ. Transient Receptor Potential Channels: Targeting Pain at the Source. Nat Rev Drug Discov (2009) 8(1):55–68. doi: 10.1038/nrd2757 19116627 PMC2755576

[14] So CL Milevskiy MJG Monteith GR. Transient Receptor Potential Cation Channel Subfamily V and Breast Cancer. Lab Invest (2020) 100(2):199–206. doi: 10.1038/s41374-019-0348-0 31822791

[15] Zhai K Liskova A Kubatka P Busselberg D. Calcium Entry Through TRPV1: A Potential Target for the Regulation of Proliferation and Apoptosis in Cancerous and Healthy Cells. Int J Mol Sci (2020) 21(11):4177. doi: 10.3390/ijms21114177 PMC7312732 32545311

[16] Hartel M di Mola FF Selvaggi F Mascetta G Wente MN Felix K. Vanilloids in Pancreatic Cancer: Potential for Chemotherapy and Pain Management. Gut (2006) 55(4):519–28. doi: 10.1136/gut.2005.073205 PMC1856157 16174661

[17] Czifra G Varga A Nyeste K Marincsak R Toth BI Kovacs I. Increased Expressions of Cannabinoid Receptor-1 and Transient Receptor Potential Vanilloid-1 in Human Prostate Carcinoma. J Cancer Res Clin Oncol (2009) 135(4):507–14. doi: 10.1007/s00432-008-0482-3 PMC12160142 18830626

[18] Weber LV Al-Refae K Wolk G Bonatz G Altmuller J Becker C. Expression and Functionality of TRPV1 in Breast Cancer Cells. Breast Cancer (Dove Med Press) (2016) 8:243–52. doi: 10.2147/BCTT.S121610 PMC5167528 28008282

[19] Stock K Kumar J Synowitz M Petrosino S Imperatore R Smith ES. Neural Precursor Cells Induce Cell Death of High-Grade Astrocytomas Through Stimulation of TRPV1. Nat Med (2012) 18(8):1232–8. doi: 10.1038/nm.2827 PMC3480991 22820645

[20] Bode AM Cho YY Zheng D Zhu F Ericson ME Ma WY. Transient Receptor Potential Type Vanilloid 1 Suppresses Skin Carcinogenesis. Cancer Res (2009) 69(3):905–13. doi: 10.1158/0008-5472.CAN-08-3263 PMC2669313 19155296

[21] de Jong PR Takahashi N Harris AR Lee J Bertin S Jeffries J. Ion Channel TRPV1-Dependent Activation of PTP1B Suppresses EGFR-Associated Intestinal Tumorigenesis. J Clin Invest (2014) 124(9):3793–806. doi: 10.1172/JCI72340 PMC4151223 25083990

[22] Yang Y Guo W Ma J Xu P Zhang W Guo S. Downregulated TRPV1 Expression Contributes to Melanoma Growth *via* the Calcineurin-ATF3-P53 Pathway. J Invest Dermatol (2018) 138(10):2205–15. doi: 10.1016/j.jid.2018.03.1510 29580868

[23] Huang J Liu J Qiu L. Transient Receptor Potential Vanilloid 1 Promotes EGFR Ubiquitination and Modulates EGFR/MAPK Signalling in Pancreatic Cancer Cells. Cell Biochem Funct (2020) 38(4):401–8. doi: 10.1002/cbf.3483 31907951

[24] Pecze L Josvay K Blum W Petrovics G Vizler C Olah Z. Activation of Endogenous TRPV1 Fails to Induce Overstimulation-Based Cytotoxicity in Breast and Prostate Cancer Cells But Not in Pain-Sensing Neurons. Biochim Biophys Acta (2016) 1863(8):2054–64. doi: 10.1016/j.bbamcr.2016.05.007 27180305

[25] Athanasiou A Smith PA Vakilpour S Kumaran NM Turner AE Bagiokou D. Vanilloid Receptor Agonists and Antagonists are Mitochondrial Inhibitors: How Vanilloids Cause Non-Vanilloid Receptor Mediated Cell Death. Biochem Biophys Res Commun (2007) 354(1):50–5. doi: 10.1016/j.bbrc.2006.12.179 17214968

[26] De La Chapa J Valdez M Ruiz F Gonzales K Mitchell W McHardy SF. Synthesis and SAR of Novel Capsazepine Analogs With Significant Anti-Cancer Effects in Multiple Cancer Types. Bioorg Med Chem (2019) 27(1):208–15. doi: 10.1016/j.bmc.2018.11.040 30528162

[27] Nur G Naziroglu M Deveci HA. Synergic Prooxidant, Apoptotic and TRPV1 Channel Activator Effects of Alpha-Lipoic Acid and Cisplatin in MCF-7 Breast Cancer Cells. J Recept Signal Transduct Res (2017) 37(6):569–77. doi: 10.1080/10799893.2017.1369121 28849985

[28] Zheng L Chen J Ma Z Liu W Yang F Yang Z. Capsaicin Enhances Anti-Proliferation Efficacy of Pirarubicin *via* Activating TRPV1 and Inhibiting PCNA Nuclear Translocation in 5637 Cells. Mol Med Rep (2016) 13(1):881–7. doi: 10.3892/mmr.2015.4623 26648574

[29] Kosar PA Naziroglu M Ovey IS Cig B. Synergic Effects of Doxorubicin and Melatonin on Apoptosis and Mitochondrial Oxidative Stress in MCF-7 Breast Cancer Cells: Involvement of TRPV1 Channels. J Membr Biol (2016) 249(1-2):129–40. doi: 10.1007/s00232-015-9855-0 26525975

[30] Deveci HA Naziroglu M Nur G. 5-Fluorouracil-Induced Mitochondrial Oxidative Cytotoxicity and Apoptosis Are Increased in MCF-7 Human Breast Cancer Cells by TRPV1 Channel Activation But Not Hypericum Perforatum Treatment. Mol Cell Biochem (2018) 439(1-2):189–98. doi: 10.1007/s11010-017-3147-1 28795251

[31] Marincsak R Toth BI Czifra G Marton I Redl P Tar I. Increased Expression of TRPV1 in Squamous Cell Carcinoma of the Human Tongue. Oral Dis (2009) 15(5):328–35. doi: 10.1111/j.1601-0825.2009.01526.x 19320840

[32] Zhang F Challapalli SC Smith PJ. Cannabinoid CB(1) Receptor Activation Stimulates Neurite Outgrowth and Inhibits Capsaicin-Induced Ca(2+) Influx in an *In Vitro* Model of Diabetic Neuropathy. Neuropharmacology (2009) 57(2):88–96. doi: 10.1016/j.neuropharm.2009.04.017 19501110

[33] Everaerts W Gees M Alpizar YA Farre R Leten C Apetrei A. The Capsaicin Receptor TRPV1 is a Crucial Mediator of the Noxious Effects of Mustard Oil. Curr Biol (2011) 21(4):316–21. doi: 10.1016/j.cub.2011.01.031 21315593

[34] Gavva NR Tamir R Klionsky L Norman MH Louis JC Wild KD. Proton Activation Does Not Alter Antagonist Interaction With the Capsaicin-Binding Pocket of TRPV1. Mol Pharmacol (2005) 68(6):1524–33. doi: 10.1124/mol.105.015727 16135784

[35] Wyatt MD Wilson DM. Participation of DNA Repair in the Response to 5-Fluorouracil. Cell Mol Life Sci (2009) 66(5):788–99. doi: 10.1007/s00018-008-8557-5 PMC2649968 18979208

[36] Damia G Broggini M. Platinum Resistance in Ovarian Cancer: Role of DNA Repair. Cancers (Basel) (2019) 11(1):119. doi: 10.3390/cancers11010119 PMC6357127 30669514

[37] Vancevska A Douglass KM Pfeiffer V Manley S Lingner J. The Telomeric DNA Damage Response Occurs in the Absence of Chromatin Decompaction. Genes Dev (2017) 31(6):567–77. doi: 10.1101/gad.294082.116 PMC5393052 28381410

[38] Shrivastav M De Haro LP Nickoloff JA. Regulation of DNA Double-Strand Break Repair Pathway Choice. Cell Res (2008) 18(1):134–47. doi: 10.1038/cr.2007.111 18157161

[39] Nakanishi K Cavallo F Brunet E Jasin M. Homologous Recombination Assay for Interstrand Cross-Link Repair. Methods Mol Biol (2011) 745:283–91. doi: 10.1007/978-1-61779-129-1_16 PMC3261721 21660700

[40] Vriend LE Prakash R Chen CC Vanoli F Cavallo F Zhang Y. Distinct Genetic Control of Homologous Recombination Repair of Cas9-Induced Double-Strand Breaks, Nicks and Paired Nicks. Nucleic Acids Res (2016) 44(11):5204–17. doi: 10.1093/nar/gkw179 PMC4914091 27001513

[41] Bennardo N Cheng A Huang N Stark JM. Alternative-NHEJ is a Mechanistically Distinct Pathway of Mammalian Chromosome Break Repair. PloS Genet (2008) 4(6):e1000110. doi: 10.1371/journal.pgen.1000110 18584027 PMC2430616

[42] Roos WP Kaina B. DNA Damage-Induced Cell Death by Apoptosis. Trends Mol Med (2006) 12(9):440–50. doi: 10.1016/j.molmed.2006.07.007 16899408

[43] Zhang F Yang H Wang Z Mergler S Liu H Kawakita T. Transient Receptor Potential Vanilloid 1 Activation Induces Inflammatory Cytokine Release in Corneal Epithelium Through MAPK Signaling. J Cell Physiol (2007) 213(3):730–9. doi: 10.1002/jcp.21141 17508360

[44] Nishino K Tanamachi K Nakanishi Y Ide S Kojima S Tanuma S. Radiosensitizing Effect of TRPV1 Channel Inhibitors in Cancer Cells. Biol Pharm Bull (2016) 39(7):1224–30. doi: 10.1248/bpb.b16-00080 27150432

[45] Li L Chen C Chiang C Xiao T Chen Y Zhao Y. The Impact of TRPV1 on Cancer Pathogenesis and Therapy: A Systematic Review. Int J Biol Sci (2021) 17(8):2034–49. doi: 10.7150/ijbs.59918 PMC8193258 34131404

[46] Castro J Aromataris EC Rychkov GY Barritt GJ. A Small Component of the Endoplasmic Reticulum is Required for Store-Operated Ca2+ Channel Activation in Liver Cells: Evidence From Studies Using TRPV1 and Taurodeoxycholic Acid. Biochem J (2009) 418(3):553–66. doi: 10.1042/BJ20081052 19007332

[47] McGarvey LP Butler CA Stokesberry S Polley L McQuaid S Abdullah H. Increased Expression of Bronchial Epithelial Transient Receptor Potential Vanilloid 1 Channels in Patients With Severe Asthma. J Allergy Clin Immunol (2014) 133 704-712(3):e704. doi: 10.1016/j.jaci.2013.09.016 24210884

[48] Ortiz-Renteria M Juarez-Contreras R Gonzalez-Ramirez R Islas LD Sierra-Ramirez F Llorente I. TRPV1 Channels and the Progesterone Receptor Sig-1R Interact to Regulate Pain. Proc Natl Acad Sci U.S.A. (2018) 115(7):E1657–66. doi: 10.1073/pnas.1715972115 PMC5816171 29378958

[49] Guminski AD Balleine RL Chiew YE Webster LR Tapner M Farrell GC. MRP2 (ABCC2) and Cisplatin Sensitivity in Hepatocytes and Human Ovarian Carcinoma. Gynecol Oncol (2006) 100(2):239–46. doi: 10.1016/j.ygyno.2005.08.046 16213010

[50] Milosevic V Kopecka J Salaroglio IC Libener R Napoli F Izzo S. Wnt/IL-1beta/IL-8 Autocrine Circuitries Control Chemoresistance in Mesothelioma Initiating Cells by Inducing ABCB5. Int J Cancer (2020) 146(1):192–207. doi: 10.1002/ijc.32419 31107974

[51] Dlugosz-Pokorska A Pieta M Janecki T Janecka A. New Uracil Analogs as Downregulators of ABC Transporters in 5-Fluorouracil-Resistant Human Leukemia HL-60 Cell Line. Mol Biol Rep (2019) 46(6):5831–9. doi: 10.1007/s11033-019-05017-w 31741260

[52] Kubo Y Sekiya S Ohigashi M Takenaka C Tamura K Nada S. ABCA5 Resides in Lysosomes, and ABCA5 Knockout Mice Develop Lysosomal Disease-Like Symptoms. Mol Cell Biol (2005) 25(10):4138–49. doi: 10.1128/MCB.25.10.4138-4149.2005 PMC1087723 15870284

[53] Ray Chaudhuri A Callen E Ding X Gogola E Duarte AA Lee JE. Replication Fork Stability Confers Chemoresistance in BRCA-Deficient Cells. Nature (2016) 535(7612):382–7. doi: 10.1038/nature18325 PMC4959813 27443740

[54] Stronach EA Cunnea P Turner C Guney T Aiyappa R Jeyapalan S. The Role of Interleukin-8 (IL-8) and IL-8 Receptors in Platinum Response in High Grade Serous Ovarian Carcinoma. Oncotarget (2015) 6(31):31593–603. doi: 10.18632/oncotarget.3415 PMC4741626 26267317

[55] Dabkeviciene D Jonusiene V Zitkute V Zalyte E Grigaitis P Kirveliene V. The Role of Interleukin-8 (CXCL8) and CXCR2 in Acquired Chemoresistance of Human Colorectal Carcinoma Cells HCT116. Med Oncol (2015) 32(12):258. doi: 10.1007/s12032-015-0703-y 26519257

[56] Zhai J Shen J Xie G Wu J He M Gao L. Cancer-Associated Fibroblasts-Derived IL-8 Mediates Resistance to Cisplatin in Human Gastric Cancer. Cancer Lett (2019) 454:37–43. doi: 10.1016/j.canlet.2019.04.002 30978440

[57] Chen S Rehman SK Zhang W Wen A Yao L Zhang J. Autophagy is a Therapeutic Target in Anticancer Drug Resistance. Biochim Biophys Acta (2010) 1806(2):220–9. doi: 10.1016/j.bbcan.2010.07.003 20637264

[58] Piya S Kornblau SM Ruvolo VR Mu H Ruvolo PP McQueen T. Atg7 Suppression Enhances Chemotherapeutic Agent Sensitivity and Overcomes Stroma-Mediated Chemoresistance in Acute Myeloid Leukemia. Blood (2016) 128(9):1260–9. doi: 10.1182/blood-2016-01-692244 PMC5009514 27268264

[59] Li J Hou N Faried A Tsutsumi S Takeuchi T Kuwano H. Inhibition of Autophagy by 3-MA Enhances the Effect of 5-FU-Induced Apoptosis in Colon Cancer Cells. Ann Surg Oncol (2009) 16(3):761–71. doi: 10.1245/s10434-008-0260-0 19116755

[60] Liu D Yang Y Liu Q Wang J. Inhibition of Autophagy by 3-MA Potentiates Cisplatin-Induced Apoptosis in Esophageal Squamous Cell Carcinoma Cells. Med Oncol (2011) 28(1):105–11. doi: 10.1007/s12032-009-9397-3 20041317

[61] Amantini C Farfariello V Cardinali C Morelli MB Marinelli O Nabissi M. The TRPV1 Ion Channel Regulates Thymocyte Differentiation by Modulating Autophagy and Proteasome Activity. Oncotarget (2017) 8(53):90766–80. doi: 10.18632/oncotarget.21798 PMC5710883 29207602

