# Supplementary material for: Transient Receptor Potential Cation Channel Subfamily V Member 1 Expression Promotes Chemoresistance in Non-Small-Cell Lung Cancer
Source: Front Oncol. 2022 Mar 25;12:773654. doi: 10.3389/fonc.2022.773654 (PMC8990814; doi:10.3389/fonc.2022.773654)
Supplement: Supplementary file 1 [file DataSheet_1.pdf]

Supplemental Information

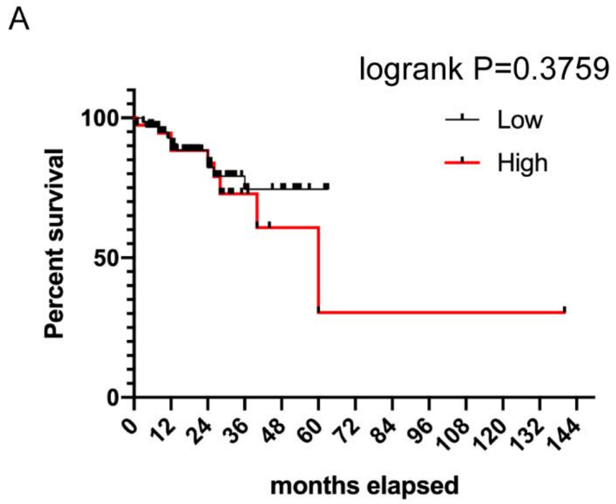

Figure S1. Overall survival of patients determined by the immunoreactivity of TRPV1. Patients with high TRPV1 expression have a trend toward poor prognosis (*P*=0.3759).

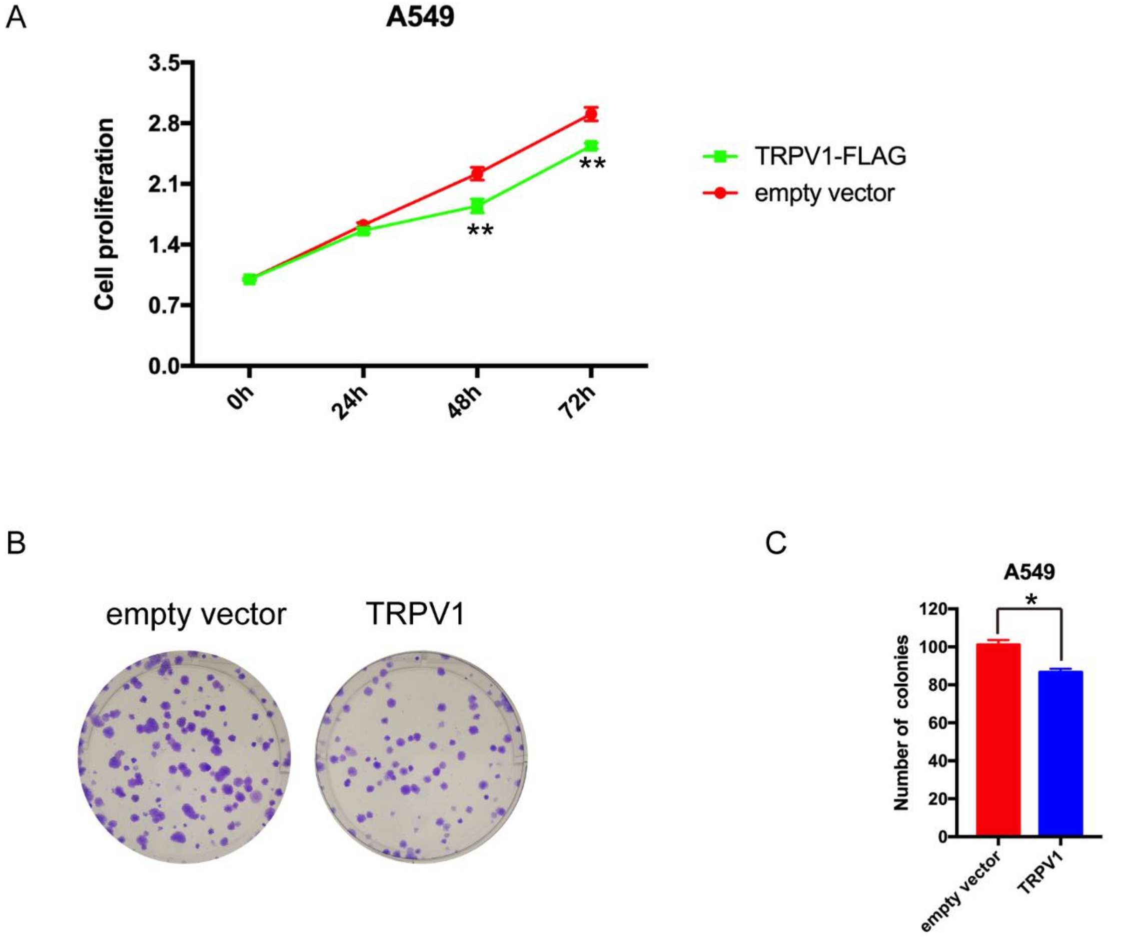

Figure S2. Overexpression of *TRPV1* suppressed NSCLC cells proliferation. A. CCK-8 analysis of the effect of *TRPV1* overexpression on A549 cells proliferation. *TRPV1* overexpression inhibited proliferation of A549 cells. The A549-empty vector and A549-TRPV1-FLAG stable cells were seeded and measured absorbance at time point of o, 24, 48 and 72 hours using CCK-8. **B.** Representative image of the effect of *TRPV1* overexpression on A549 cells proliferation was determined by colony formation assay. *TRPV1* overexpression suppressed A549 cells clonogenic capacity. **C.** statistical analysis of B. Data represent mean±S.E. *$P < 0.05$. **$P < 0.01$.

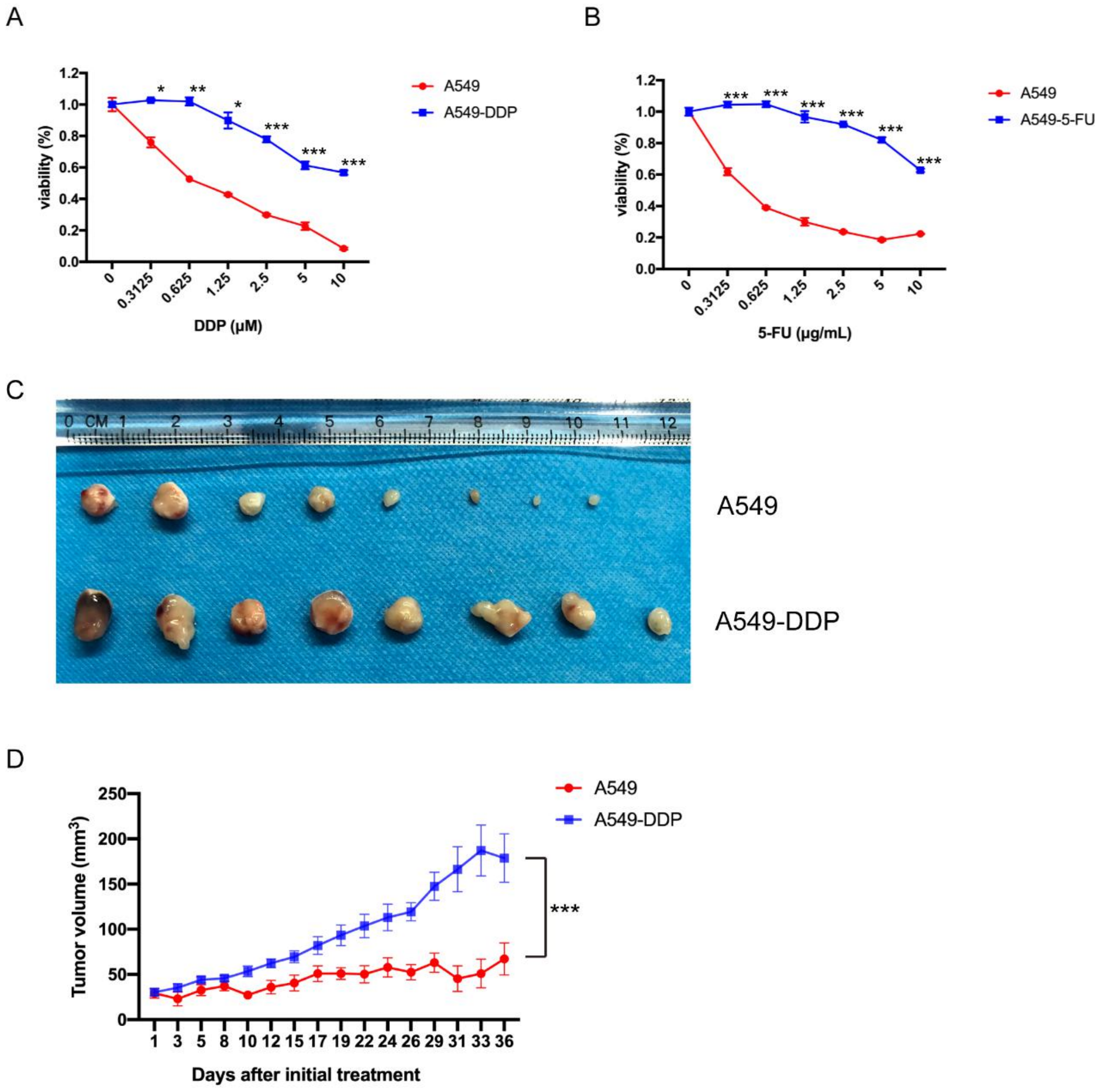

Figure S3. The resistance characteristics of A549-DDP/5-FU resistant cell lines. A. A549-DDP resistant cells showed DDP resistance. **B.** The A549-5-FU resistant cells showed 5-FU resistance. **C.** Images of A549 and A549-DDP xenografts after DDP treatment. **D.** Tumor volume of animals with A549 and A549-DDP xenografts. Data represent mean±SEM; **P* < 0.05. ***P* < 0.01. ****P* < 0.001.

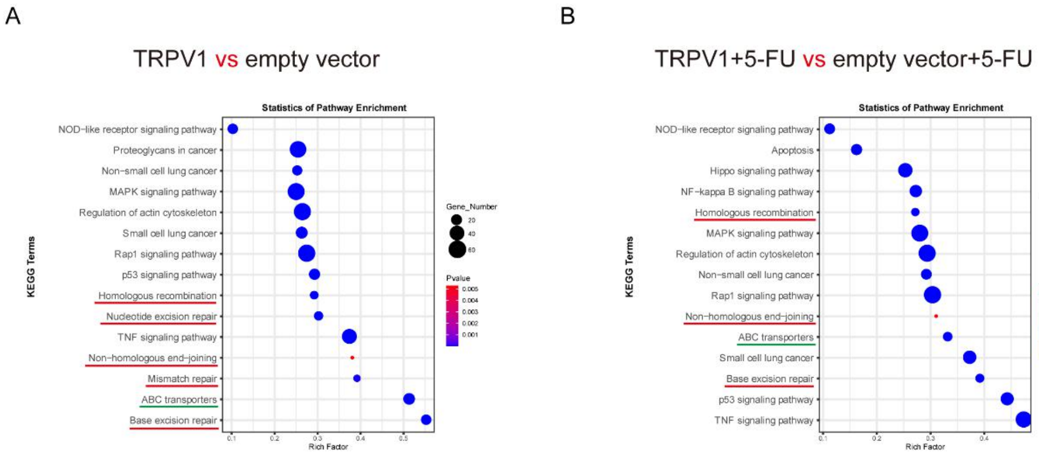

Figure S4. KEGG pathway analysis of differentially expressed genes. A. TRPV1 overexpression vs. empty vector transfection: Advanced bubble chart showing enrichment of differentially expressed genes in signaling pathways. **B.** Advanced bubble chart shows enrichment of differentially expressed genes of TRPV1 overexpression vs. empty vector transfection in the presence of 5-FU in signaling pathways. The bubble size denotes the number of differentially expressed genes enriched in the pathway. The color of the bubble represents the *P*-value range.

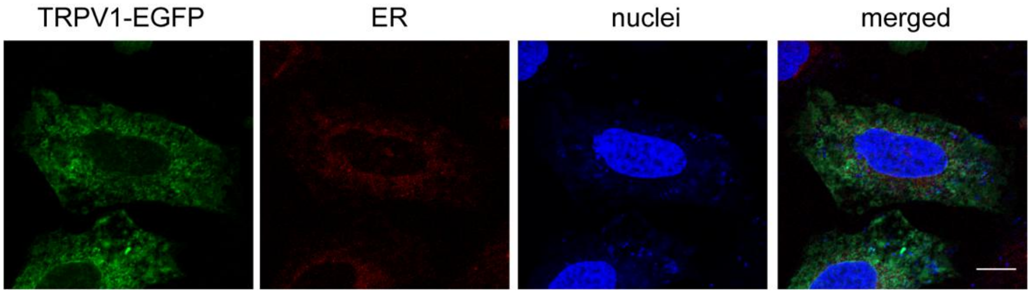

Figure S5. TRPV1-EGFP localized in endoplasmic reticulum (ER). Representative confocal microscopic images of TRPV1 and ER colocalization in A549 cells. ER was stained with ER-tracker. The cell nuclei were stained with DAPI. Scale bar, 10 μm.

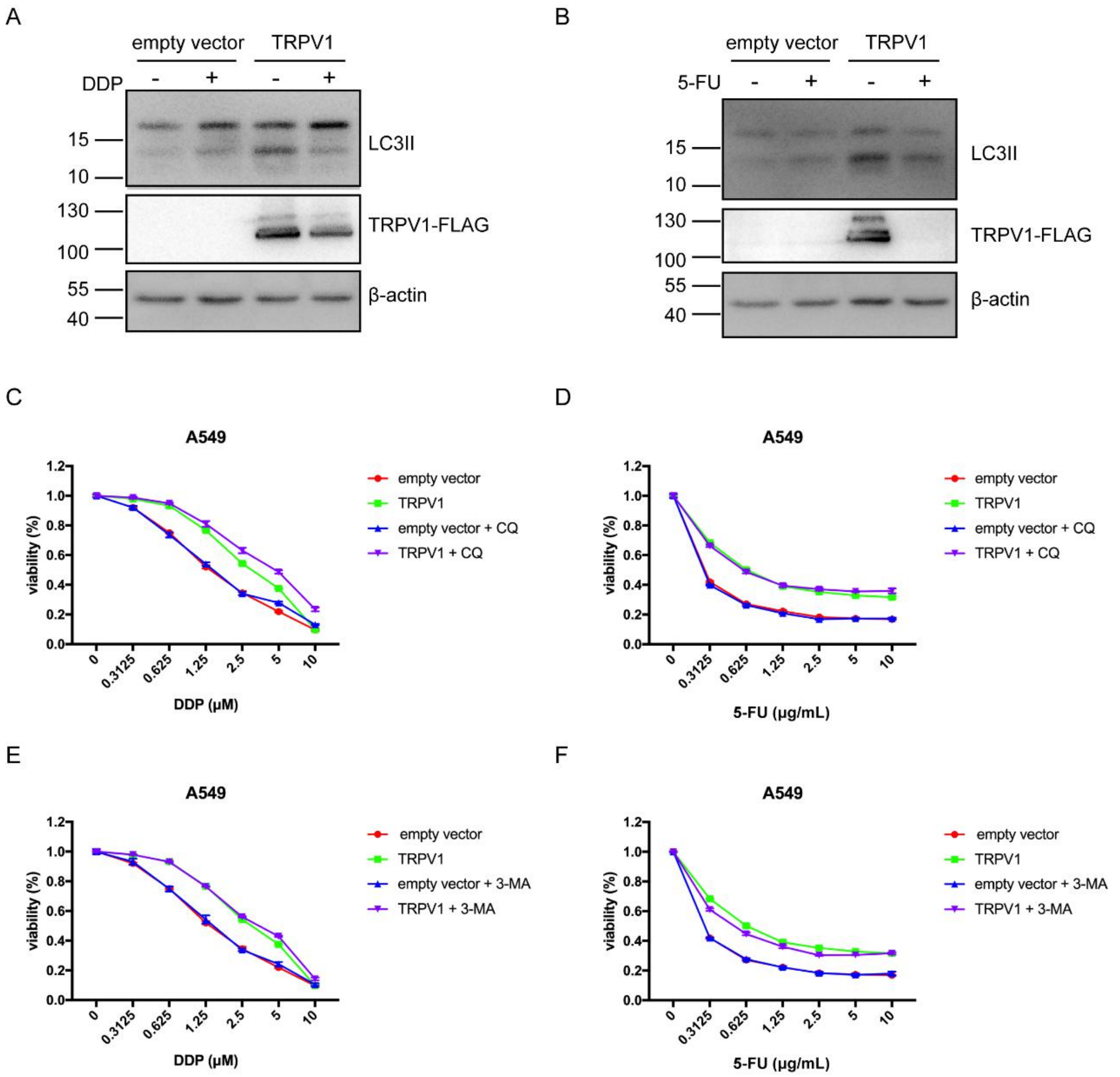

Figure S6. The DDP and 5-FU resistance induced by *TRPV1* overexpression was not due to stimulation of autophagy. A. Western blot analysis showing increased LC3II protein levels in A549 cells overexpressing *TRPV1* and treated with DDP (1 μM) for 24 h; β-actin served as the loading control. **B.** Western blot analysis showing increased LC3II protein levels in A549 cells overexpressing *TRPV1* and treated with 5-FU (0.5 μg/mL) for 24 h; β-actin served as the loading control. **C.** CCK-8 assay showing that treatment with the autophagy inhibitor CQ (10 μM) for 72 h had no effect on the TRPV1-mediated DDP resistance of A549 cells overexpressing *TRPV1*. **D.** CCK-8 assay showing that treatment with the autophagy inhibitor CQ (10 μM) for 72 h had no effect on the TRPV1-mediated 5-FU resistance of A549 cells overexpressing *TRPV1*. **E.** CCK-8 assay showing that treatment with the autophagy inhibitor 3-MA (10 μM) for 72 h had no effect on the TRPV1-mediated DDP resistance of A549 cells overexpressing *TRPV1*. **F.** CCK-8 assay showing that treatment with the autophagy inhibitor 3-MA (10 μM) for 72 h had no effect on TRPV1-mediated 5-FU resistance of A549 cells overexpressing *TRPV1*. Data represent mean±SEM.
